# Formation of the Embryonic Organizer Is Restricted by the Competitive Influences of Fgf Signaling and the SoxB1 Transcription Factors

**DOI:** 10.1371/journal.pone.0057698

**Published:** 2013-02-28

**Authors:** Cheng-Liang Kuo, Chi Man Lam, Jane E. Hewitt, Paul J. Scotting

**Affiliations:** Centre for Genetics and Genomics, School of Biology, University of Nottingham, QMC, Nottingham, United Kingdom; Texas A&M University, United States of America

## Abstract

The organizer is one of the earliest structures to be established during vertebrate development and is crucial to subsequent patterning of the embryo. We have previously shown that the SoxB1 transcription factor, Sox3, plays a central role as a transcriptional repressor of zebrafish organizer gene expression. Recent data suggest that Fgf signaling has a positive influence on organizer formation, but its role remains to be fully elucidated. In order to better understand how Fgf signaling fits into the complex regulatory network that determines when and where the organizer forms, the relationship between the positive effects of Fgf signaling and the repressive effects of the SoxB1 factors must be resolved. This study demonstrates that both *fgf3* and *fgf8* are required for expression of the organizer genes, *gsc* and *chd*, and that SoxB1 factors (Sox3, and the zebrafish specific factors, Sox19a and Sox19b) can repress the expression of both *fgf3* and *fgf8*. However, we also find that these SoxB1 factors inhibit the expression of *gsc* and *chd* independently of their repression of *fgf* expression. We show that ectopic expression of organizer genes induced solely by the inhibition of SoxB1 function is dependent upon the activation of *fgf* expression. These data allow us to describe a comprehensive signaling network in which the SoxB1 factors restrict organizer formation by inhibiting Fgf, Nodal and Wnt signaling, as well as independently repressing the targets of that signaling. The organizer therefore forms only where Nodal-induced Fgf signaling overlaps with Wnt signaling and the SoxB1 proteins are absent.

## Introduction

The embryonic organizer, as defined by the experiments of Spemann and Mangold, is one of the earliest and most critical patterning structures of vertebrate development [1]. Although several of the signals and genes involved in organizer formation have been identified, our understanding of the processes that control its formation is far from complete.

We have previously shown that the SoxB1 family of transcription factors can repress multiple genes associated with organizer formation. This family comprises *sox1*, *sox2* and *sox3* and the zebrafish specific genes, *sox19a* and *sox19b* [2]. Only *sox3*, *sox19a* and *sox19b* are expressed in zebrafish at the time of organizer formation. Recent work has implicated Fgf signaling as a key positive regulator in organizer formation in zebrafish [3]. Given the strong inducing effects of Fgf signaling and the reciprocal strong repressive effects of Sox3, elucidating how these opposing forces interact is crucial to our understanding of organizer formation.

Fgf signaling has been shown to promote Sox3 expression in several developmental contexts [4], [5], [6], but this does not appear to be true at the earliest stages of development when organizer formation occurs [7]. On the other hand, Sox2 has been shown to regulate the expression of *fgf4* [8], so there is a precedent to suggest that SoxB1 factors could act upstream of *fgf* gene expression.

We have shown previously that the central role played by the soxB1 factors in restricting organizer formation is achieved both by inhibiting Nodal signaling and directly repressing the gene targets of Wnt signaling [9]. Conversely, inhibition of SoxB1 protein function is sufficient to induce expression of Nodal-related signals and organizer genes in the animal pole of early embryos with consequent axis duplications.

This study set out to confirm the role of Fgf signaling in organizer formation and to establish how it fits into the network of factors in which Sox3 acts as a central repressor. In particular, we aimed to establish whether the repressive effects of sox3 on organizer formation could be explained by repression of Fgf signaling rather than by repression of the targets of Fgf signaling. We show that *fgf3* and *fgf8* are necessary for the expression of the organizer genes, *gsc* and *chd*. The SoxB1 factors can repress both *fgf3* and *fgf8* expression in addition to directly repressing Fgf target genes. However, Fgf signaling does not reciprocally repress *soxB1* expression in the region of the organizer. Inhibition of SoxB1 function resulted in ectopic expression of the *fgfs*, and this Fgf activity was required for the ectopic activation of other organizer genes. These data reveal a complex network of signaling events that promote organizer formation, with the expression of every component of that network being repressed by Sox3 (and by Sox19a and Sox19b). We propose a model in which the organizer forms only where Fgf signaling is sufficiently high and the SoxB1 factors are absent.

## Results

### 1. SoxB1 Factors Repress the Expression of *fgf3* and *fgf8* in the Organizer

Amongst the Fgf family, *fgf3* and *fgf8* are expressed specifically in the organizer in zebrafish [10]. *fgf3* and *fgf8* transcripts could first be detected in the dorsal region of the forming organizer at about 4.5 hpf, about 1 hr after expression of the earliest marker of the organizer, *boz*, is first seen (see Furthauer et al. 2001 [10] and Fig. S1 in the supplementary material).

**Figure 1:**
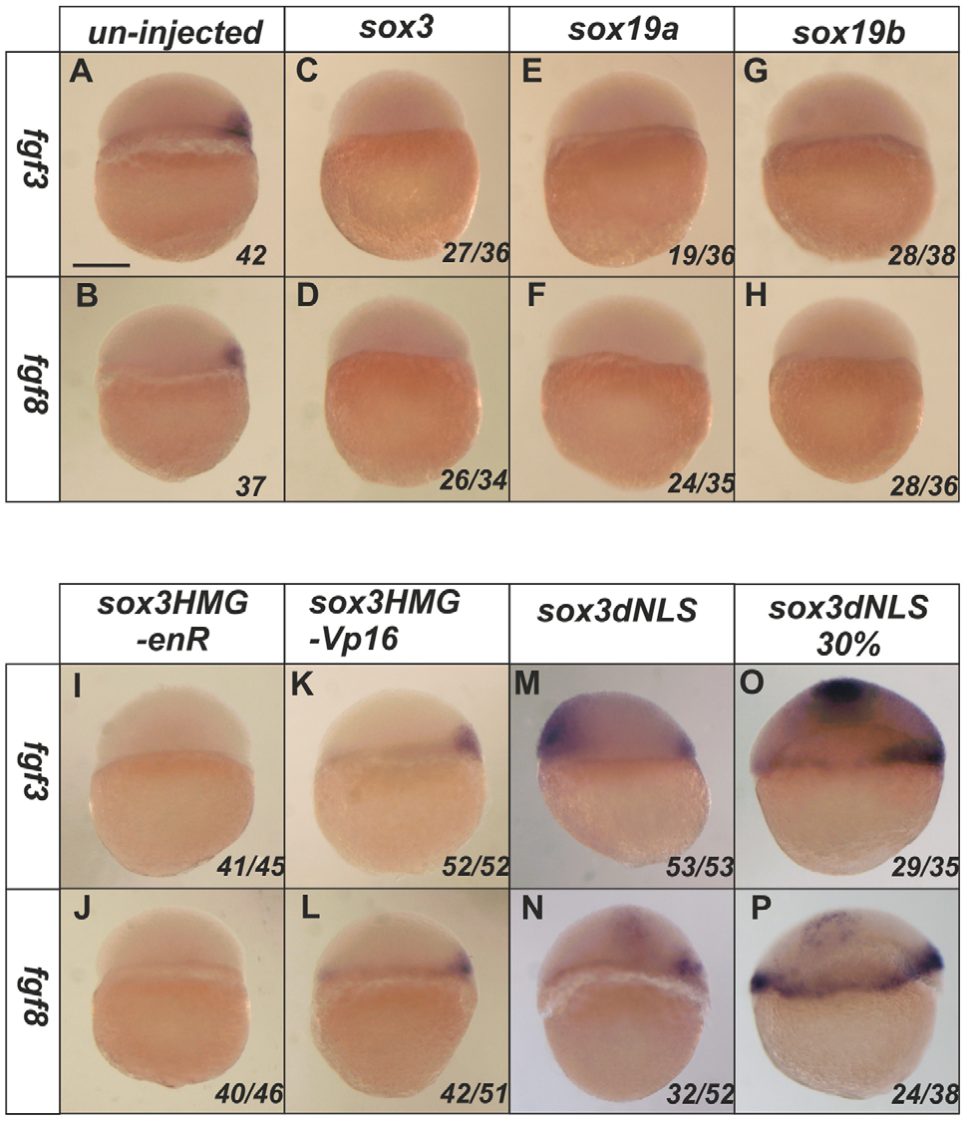
SoxB1 factors acts as transcriptional repressors to inhibit the expression of *fgf3* and *fgf8.* At 4.5 hpf, the expression of *fgf3* and *fgf8* is restricted in the dorsal shield region of un-injected embryos (**A–B**). Injection of *sox3, sox19a* or *sox19b* RNA at the 1–2 cell stage caused complete loss of expression of both *fgf3* and *fgf8* at 4.5 hpf (**C–H**). A Sox3HMG-EnR (**I**,**J**) but not a Sox3HMG-VP16 (**K**,**L**) fusion mimicked the function of wild-type Sox3 to inhibit *fgf3* and *fgf8* expression. Ectopic expression of *fgf3* and *fgf8* was induced by the dnSox3 construct injected at the 1–2 cell stage and analysed at 4.5 hpf (**M**,**N**) or later at 30% epiboly (5.5 hpf) (**O**,**P**). All images are lateral views with dorsal to the right (where this can be determined). The proportion of embryos exhibiting these phenotypes is shown at the bottom right of each panel. Scale bar in panel **A** represents approximately 100 µm.

We first examined whether members of the SoxB1 subfamily could affect expression of these *fgfs* during the period of organizer induction. Injection of *sox3, sox19a* or *sox19b* RNA at the 1–2 cell stage resulted in a complete loss of expression of both *fgf3* and *fgf8* in the organizer (Fig. 1A–H). We found that RNA encoding a Sox3HMG-enR fusion protein, but not the constitutive activator Sox3HMG -VP16, was able to mimic the ability of the SoxB1 factors to repress the expression of both *fgf3* and *fgf8* (Fig. 1I–L). Hence, it appears that the SoxB1 factors are likely to repress the *fgf* genes directly.

We next investigated whether inhibition of SoxB1 function would be sufficient to elicit ectopic expression of the *fgfs* in the absence of any other additional dorsal or vegetal signals. For this, a dominant negative approach is preferable to a morpholino (MO) knockdown approach. Because of redundancy between different members of the SoxB1 family and maternal expression of at least one of the family members [11], [9], a phenotype is only seen in morpholino injected embryos at stages of development after organizer formation (as reported by Okuda et al. 2010 [11]) and no effect on the early expression of the *fgfs* was seen (data not shown). This suggest that there is sufficient maternal protein for at least one of the SoxB1 factors to mask any effects of blocking translation of the other factors. However, we have previously shown that a dominant negative Sox3 construct, in which the nuclear localization signals were mutated (hereafter referred to as dnSox3) interferes with the activity of all the SoxB1 factors (by inhibiting their nuclear localization), and was able to elicit ectopic expression of four organizer markers (*boz, sqt, gsc* and *chd*), an effect rescued by co-injection with any of the SoxB1 factors [9]. Here, we found that injection of the same dnSox3 construct also induced ectopic expression of both *fgf3* and *fgf8* at 4.5 hpf in a manner similar to the induction of other markers of organizer (Fig. 1M,N). This effect was more striking at 30% epiboly (approximately 5 hpf), a stage when endogenous *fgf* expression is more robust (Fig. 1O,P). One concern in using dominant-negative approaches is that the dnSox3 construct might not only block the function of the protein of interest, but might also generate unrelated neomorphic effects. However, in this case, like the effects on other markers of the organizer, this induction of fgf expression by dnSox3 was rescued by overexpression of *sox3*, *sox19a* or *sox19b* with the dnSox3 similarly negating the ability of any of the SoxB1 factors from repressing *fgf* expression and resulting in reversion to wild type *fgf* expression (see Fig. S2 in the supplementary material). This rescue experiment indicates that the effects of the dnSox3 described are via inhibiting SoxB1 function and are not neomorphic effects. Together these data indicate that the endogenous SoxB1 proteins repress the expression of *fgf3* and *fgf8* in the organizer and that interfering with this repression using a dnSoxB1 is sufficient to elicit ectopic expression of these *fgf* genes in sites distant from the organizer.

### 2. Sox3 Binds Directly to an Evolutionarily Conserved Element in the Promoter Region of *fgf3*

**Figure 2:**
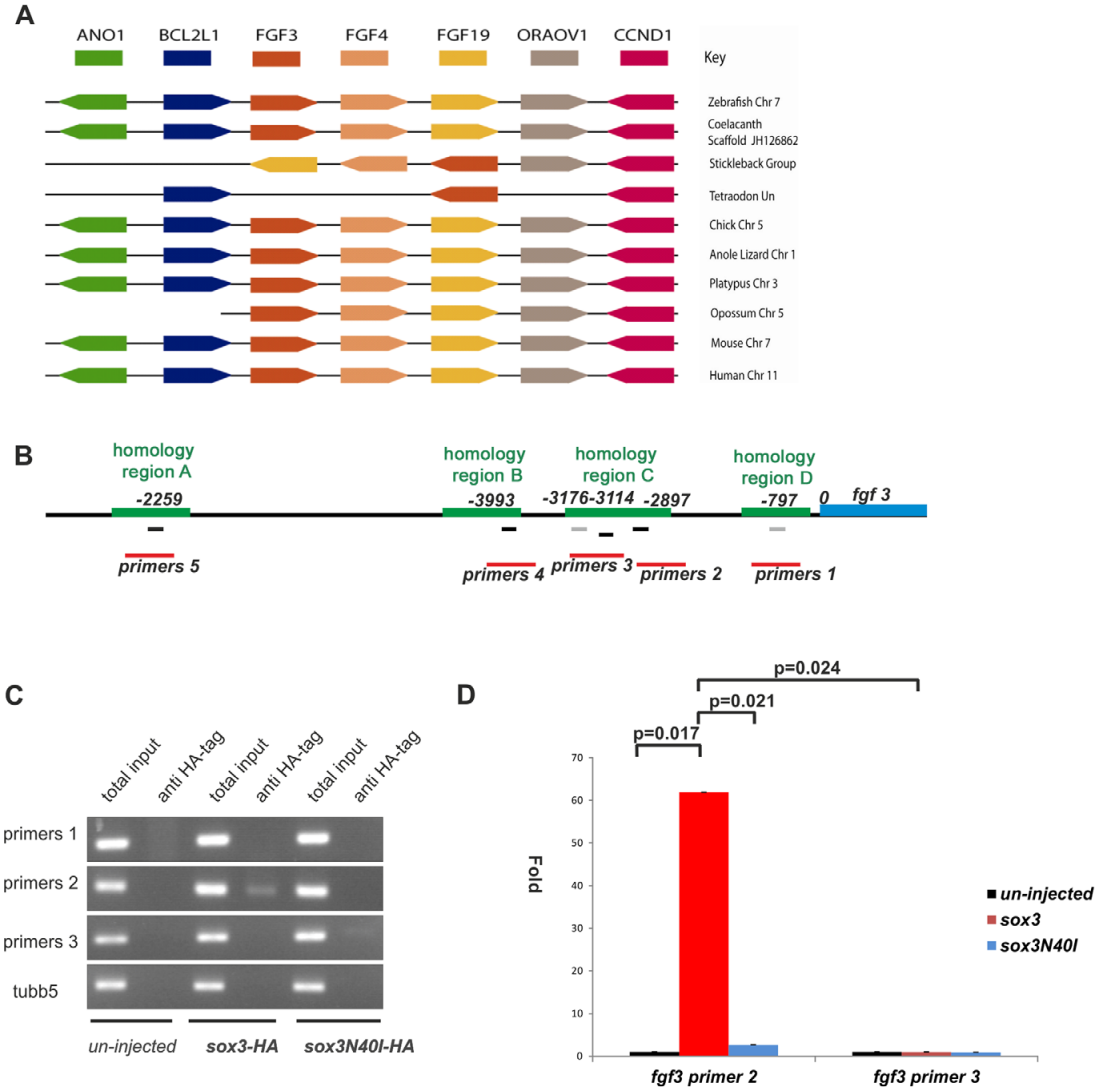
Sox3 can directly bind to a region upstream of *fgf3*. (**A**) Synteny in the region of the genome flanking the *fgf3* gene. Coloured boxes indicate different genes and direction of transcription. Not to scale. Absence of line indicates incomplete genomic scaffold information. (**B**) Diagram showing the upstream region of *fgf3*. Green bars indicate regions of homology among different species. The position of potential Sox binding sites (A/T)(A/T)CAA(A/T)G within these homologous regions are shown as black bars and similar potential Sox binding sites lacking the final 3′ “G” are shown as gray bars. The red bars show the PCR products, including the Sox binding sites that would be produced by different primer pairs. (**C**) 25 pg *sox3*-HA and *sox3*N40I-HA RNA were injected at the 1–2 cell stage embryos and harvested at 4.5 hpf. ChIP analysis using an anti-HA antibody to precipitate Sox3 and bound DNA. PCR results after ChIP procedure showed that the DNA fragments pulled down by Sox3-HA can be amplified only by primer pair 2. *tubb5* was included as a negative control. (**D**) Quantitative PCR results of precipitated chromatin using primer pairs 2 and 3 showed that the target sequence for primer pair 2 was significantly enriched following IP of WT Sox3 whereas the target for primer pair 3 was not. Values represented as fold change compared to the uninjected value.

In order to investigate whether *fgfs* could be direct targets of the SoxB1 transcription factors, we analysed binding of Sox3 to the *fgf3* promoter using ChIP-PCR. Comparison of genome organization between species in the region of *fgf3* using the ENSEMBL database showed that a significant degree of synteny had been retained across a wide diversity of species from coelacanth to human, including zebrafish (Fig. 2A). This allowed us to identify regions around the *fgf3* transcription unit suitable for comparison to find conserved potential regulatory sequences. Previous studies have found that highly conserved non-coding sequences 5′ to a gene often harbour functional transcription factor binding sites [12]. We therefore used PipMaker [13] to indentify such regions of conservation. This identified two regions (regions ‘C’ and ‘D’ in Fig. 2B) that were highly conserved between fish, birds, lizard, frogs, platypus and opposum and a third region only conserved among fish (region ‘B’ in Fig. 2B) within 4 kb of the *fgf3* transcription start site (TSS). A fourth region (region ‘A’ in Fig. 2B) positioned approximately 23 Kb upstream of the TSS, was also highly conserved including in mouse and human (see Fig. S3 in the supplementary material). All of these regions contained potential Sox-binding sites (Fig. 2B and see Fig. S4 in the supplementary material for full sequence comparisons). We therefore designed primers to detect all of these regions for use in ChIP-PCR experiments (as labelled in Fig. 2B).

Since commercially available antibodies were unable to immunoprecipitate endogenous Sox3, we used ectopic expression of an HA-tagged version of Sox3. As in our previous study [9], in order to avoid non-specific Sox3-DNA interactions, we injected an amount of RNA that produced protein at a level below that of the endogenous protein at 30% epiboly (analysed by Western blot, data not shown). HA antibody immunoprecipitation (IP) on uninjected embryos and IP of an HA-tagged N40I DNA-binding mutant of Sox3 [9] were included as additional negative controls. The HA antibody did not precipitate a detectable level of any of the *fgf3* target regions in uninjected embryos (Fig. 2C,D). Similarly, even in fish injected with HA-tagged Sox3, there was no detectable precipitation of *fgf3* fragments A, B or D or a *tubulin* control fragment (*tubb5*) (Fig. 2C *)*. However, pull down of the HA-tagged Sox3 did result in robust detection of the proximal region (primer pair 2) of *fgf3* fragment C (Fig. 2C). qPCR verified that the amount of the proximal end of region C (primer pair 2) precipitated in Sox3 injected embryos was significantly more than was precipitated following injection of the N40I DNA-binding mutant, or the amount of the distal end of fragment C (primer pair 3) that was precipitated (Fig. 2D).

These data demonstrate that, despite the presence of Sox binding consensus sequences in all four conserved regions upstream of *fgf3*, Sox3 only binds to the proximal end of region C, 2888 bp upstream of the *fgf3* TSS.

### 3. Induction of the Organizer Genes, *gsc* and *chd,* by Inhibition of SoxB1 Function Requires Fgf Signaling

Inhibition of SoxB1 factors can induce the ectopic expression of *gsc* and *chd*, which is normally restricted to the organizer [9], and *fgf3* and *fgf8* can now be added to that list of genes repressed by the SoxB1s. Since Fgfs normally play a central role in the expression of the other organizer genes, the ectopic expression of organizer genes when SoxB1 function is repressed might require Fgf signaling. Alternatively, in the absence of repression by the SoxB1 factors, Fgf signaling might no longer be necessary for the expression of *gsc* and *chd*.

**Figure 3:**
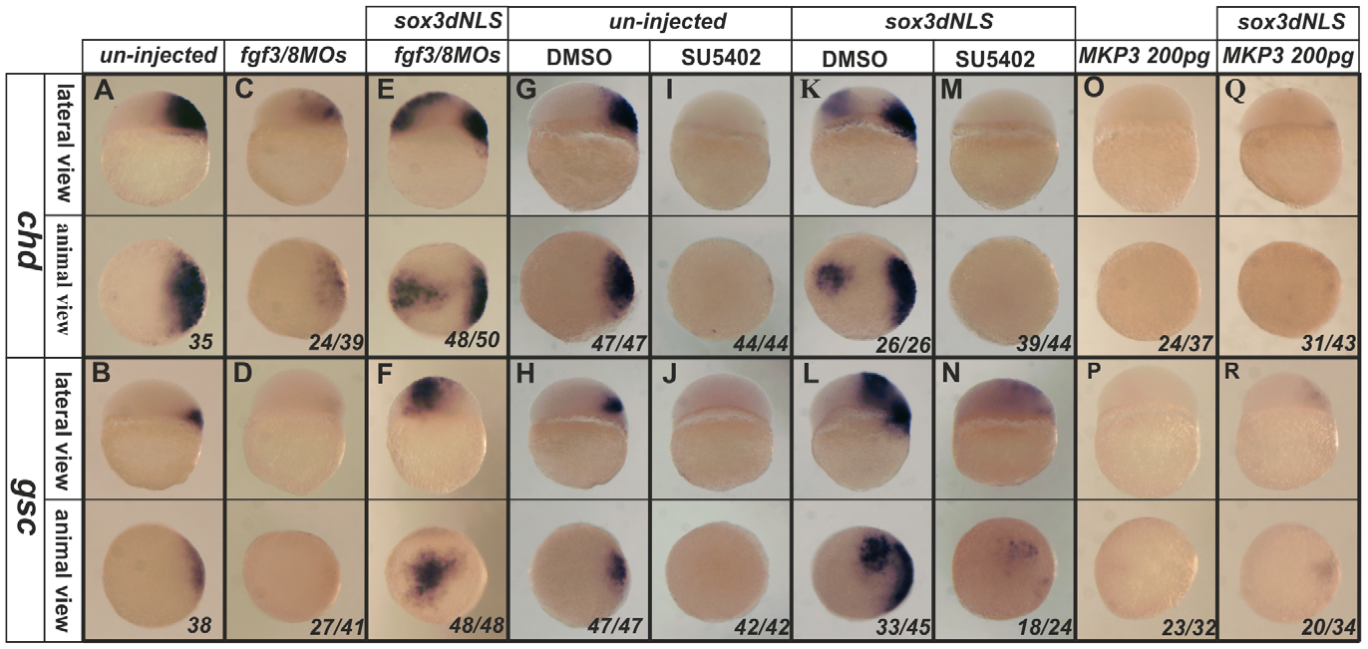
Induction of ectopic *chd* and *gsc* expression by dnSox3 **requires Fgf signalling.** Endogenous expression of *chd* and *gsc* (**A**,**B**) was inhibited by injection at 1–2 cell stage of a combination of morpholinos targeting both *fgf3* and *fgf8* (**C**,**D**), but these had little effect upon the ectopic expression of *chd* and *gsc* induced by injection of a dnSox3 construct (**E**,**F**). Treatment of embryos with the FGF signalling inhibitors, SU5402 (**I**,**J**) or MKP3 (**O**,**P**) (but not an equivalent concentration of the solvent DMSO alone) (**G**,**H**), at the 1–2 cell stage totally inhibited both endogenous expression of *chd* and *gsc* and ectopic expression induced by injection of a dnSox3 construct (**K–N**,**Q**,**R**). Lateral view and dorsal is to the right in upper panels, viewed from animal pole in lower panels. The proportion of embryos exhibiting these phenotypes is shown at the bottom right of each panel.

We first examined the role of Fgf3 and Fgf8 which have previously been implicated in organizer formation [14], [3]. Published MOs against *fgf3* and *fgf8* were used to test this. Alone, inhibition of either *fgf3* or *fgf8* expression caused substantial but incomplete repression of the endogenous expression of *gsc* or *chd* (See Fig. S5 in the supplementary material), while a combination of both MOs resulted in an even greater repression of *chd* expression and complete inhibition of *gsc* expression Fig. 3A–D).

We next asked whether the ectopic expression of *gsc* and *chd* that was induced when SoxB1 function was inhibited using a dnSox3 construct, also required the activity of these Fgfs (which, as shown above in Fig. 1O–P, are induced ectopically when SoxB1 function is inhibited). In the presence of the combined *fgf3/8* MOs, the dnSox3 was still able to elicit robust ectopic expression of these organizer genes (Fig. 3E–F). However, that fact that treatment with MOs targeting fgf3 and fgf8 did not affect the response to dnSox3 could be because MOs generally only cause knock down rather than complete loss of target proteins. Alternatively, other Fgfs (such as Fgf24 or Fgf17, which are expressed even earlier than Fgf3 and Fgf8 in mesoderm and organizer [15], [16]), may also play a role. Therefore we used the FGFR1 inhibitor, SU5402, as a more effective and broader inhibitor of Fgf signaling to address this question. Inhibition of Fgf signaling using SU5402 completely abolished both the endogenous and the ectopic induction of these *gsc* and *chd* expression by dnSox3 (Fig. 3G–N). In order to confirm that this effect was due to inhibition of Fgf signaling, we repeated this experiment using the alternative, intracellular, Fgf signaling inhibitor, MKP3 [17]. The result was identical to that seen with SU5402 (Fig. 3O–R).

Together, these data show for the first time that both Fgf3 and Fgf8 play a role in the expression of *gsc* and *chd* in the organizer, and that the induction of ectopic *fgf* expression, when SoxB1 function is inhibited, is necessary for the ectopic induction of *gsc* and *chd* expression (but not for the induction of *boz* and *sqt* expression, data not shown).

### 4. SoxB1 Factors also Act Independently of Fgf Signaling to Repress *chd* and *gsc* Expression

**Figure 4:**
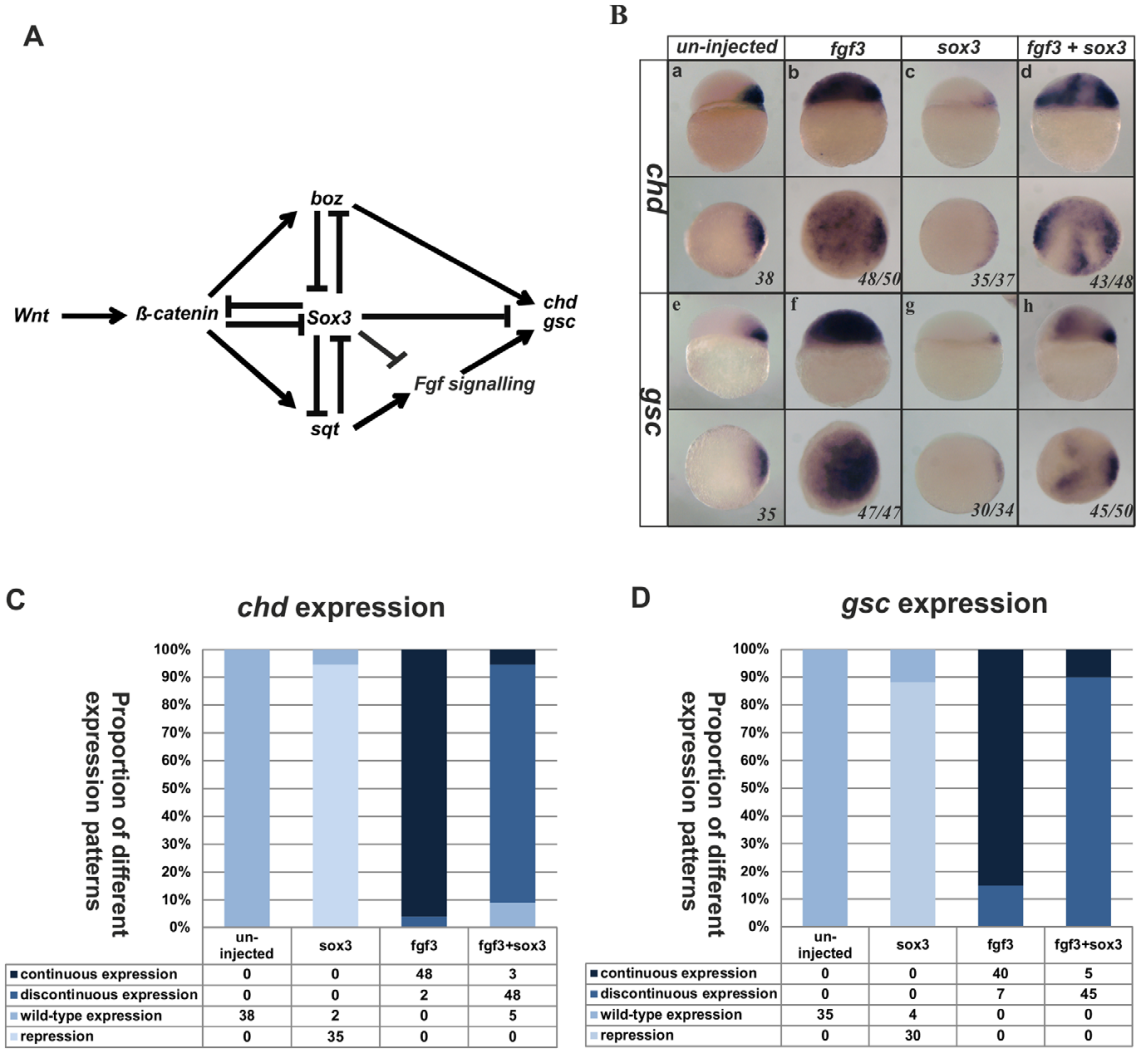
Sox3 represses expression of *chd* and *gsc* independently of repressing *fgf* expression. (**A**) Model of the signalling network that controls organizer formation. Sox3 plays a central role in this model to repress Fgf signalling in addition to independently repressing other genes needed for organizer formation. Injection of *fgf3* RNA at the 1–2 cell stage dramatically expanded both *chd* and *gsc* expression in the animal hemisphere (**Ba**,**b**,**e**,**f**). Injection of wild-type *sox3* RNA not only inhibited the endogenous expression of *chd* and *gsc* (**Bc**,**g**), but was also able to partially inhibit the expansion of *chd* and *gsc* expression that was induced by injection of *fgf3* (**Bd**,**h**). (**C**,**D**) Graphical representation of the numbers of embryos affected in these experiments. Lateral view and dorsal is to the right in upper panels, viewed from animal pole in lower panels. The proportion of embryos exhibiting these phenotypes is shown at the bottom right of each panel.

Based on the above observations, a network of signals leading to organizer formation can be proposed as shown in Fig. 4A. We have shown previously that repression of *gsc* and *chd* expression by SoxB1 factors appears to be independent of the repression of *sqt* and *boz* which are upstream activators of *gsc* and *chd* [9]. However, whether the inhibition of *gsc* and *chd* by SoxB1 factors is direct or entirely due to its inhibition of Fgf signaling required testing. In order to do this, we combined overexpression of Sox3 and Fgf3 and analysed the effect upon the expression of *gsc* and *chd*. Fgf3 overexpression alone caused a dramatic expansion of both *gsc* and *chd* expression throughout the epiblast (Fig. 4Ba,b,e,f,C,D). This is consistent with the data of Maegawa *et* *al.* (2006) who showed that Fgf3 or Fgf8 could rescue the loss of *chd* expression in mutant fish that lacked functional ß-catenin2 [3]. However, when Fgf3 and Sox3 were overexpressed together, we found that, although Fgf3 still induced *gsc* and *chd* expression over broad parts of the embryo, in the regions where Sox3 was expressed (as indicated by staining for the overexpressed HA-Sox3 protein, Fig. S6 in the supplementary material), *gsc* and *chd* expression was absent (Fig. 4Bc,d,g,h,C,D). Since the direct effects of Sox3 are cell autonomous, this is consistent with Sox3 repressing *gsc* and *chd* in the patches where it is expressed, while Fgf3 (which has a broader effect due to its extracellular diffusion) can only expand expression in those regions where cells are not over expressing Sox3. It seems, therefore, that in addition to repressing expression of the *fgfs*, Sox3 also independently represses the expression of *gsc* and *chd* downstream of Fgf signaling.

Taken together with our previous work [9], these data suggest that Sox3 represses the transcription of *gsc* and *chd* independently of its effects on *boz* expression and on Wnt, Nodal and Fgf signaling. In order to verify this, we tested the ability of Sox3 to inhibit *gsc* and *chd* expression when all of these organizer promoting factors were overexpressed together. We found that Fgf signaling caused a broader expansion (presumable because this is not limited to a cell autonomous effect) than Boz, but injection of both factors combined increased the level of ectopic *chd* expression and addition of Sqt and a constitutively active ß-catenin (S37A) caused even stronger expression of both *chd* and *gsc* throughout the epiblast (See Figs. S7A and S8A in the supplementary material). However, irrespective of the combination of organizer-promoting factors used, Sox3 was able to generate patches of the embryo in which expression was absent (See Fig. S7B and S8B in the supplementary material).

### 5. Sox3 and Fgf Signaling Regulate *chd* Independently from their Regulation of *gsc*

**Figure 5:**
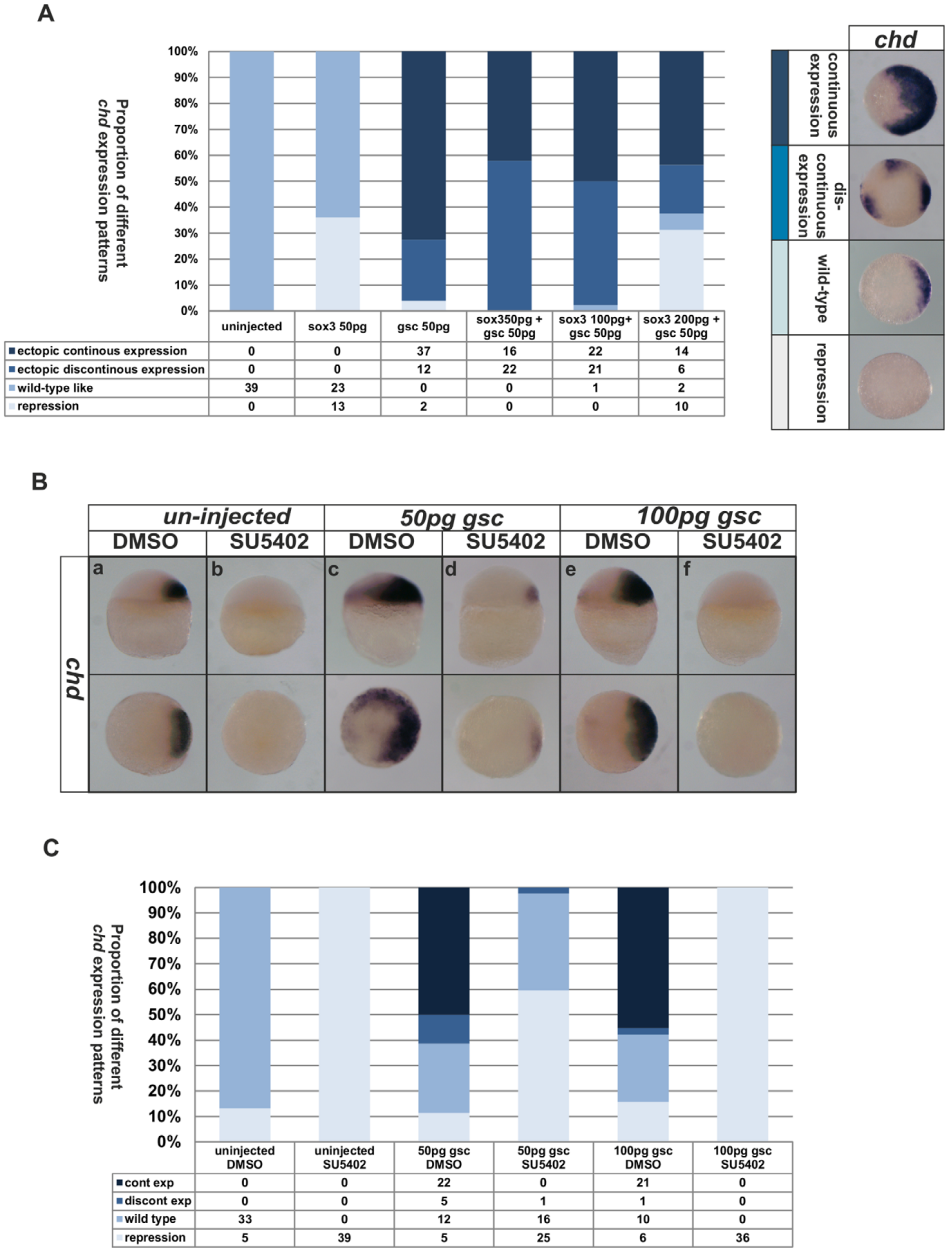
Effects of FGF and Sox3 upon the expression of *chd* cannot be rescued by Gsc. Injection of different mixtures of *sox3* or *gsc* RNA resulted in a range of expression levels of *chd* from complete repression to dramatic ‘continuous’ expansion as shown in the right of panel **A**. The phenotypes seen when combinations of *sox3* and *gsc* were injected, were intermediate between those when *sox3* or *gsc* alone were injected (Shown in bar chart in panel **A**). Treatment with SU5402 was able to inhibit *chd* expression (**Ba**,**b**) even when 50 pg (**Bc**,**d**) or 100 pg (**Be**,**f**) *gsc* RNA was also injected. Shown in bar chart in panel **C**. The proportion of embryos exhibiting these phenotypes is shown at the bottom right of each panel.

To date, we have analysed *gsc* and *chd* as two of the earliest markers of the organizer. Since *gsc* encodes a transcription factor and injected *gsc* RNA has been shown to induce *chd* expression, albeit in a non-cell autonomous manner [18], [19] we set out to determine if the effects of the Fgfs and the SoxB1 factors on *chd* are indirectly due to effects on *gsc*. We first showed that injection of RNA encoding Gsc dramatically expanded the domain of *chd* expression (Fig. 5). In order to determine if the repression of *chd* by Sox3 was indirectly through the repression of *gsc* expression, we next determined whether *gsc* RNA was able to rescue the repression of *chd* by Sox3. When *gsc* and *sox3* RNA were injected together, Sox3 appeared to repress *chd* expression in the patches of cells in which it was expressed (as shown by staining for the HA-tag it carried, data not shown) within a broader domain of *chd* expression induced (non-cell autonomously) by Gsc (Fig. 5A).

Next, in order to determine if the loss of *chd* expression seen when Fgf signaling was inhibited was indirect, due to loss of *gsc* expression, we similarly determined whether Gsc could rescue the loss of *chd* expression seen in embryos treated with SU5402 (Fig. 5B). As in the previous experiment, we found that Gsc could not rescue *chd* expression in the absence of Fgf signaling (Fig. 5B,C) indicating that Fgf signaling is independently required for both *chd* and *gsc* expression. These experiments show that, despite the fact that Gsc acts upstream of *chd* expression, both Fgf signaling and Sox3 act independently upon both genes.

Maegawa et al. (2006) have shown previously that knock down of Sqt results in loss of *fgf* expression [3]. In order to complete the picture of how Fgf signaling fits into the network of events upstream of organizer formation, we analysed to what extent Fgf signaling could compensate for loss of Sqt. We found that injection of *fgf3* RNA was able to rescue expression of both *gsc* and *chd* when Sqt was knocked down (See Fig. S9 in the supplementary material), implying that the only requirement for Sqt in regulating these genes is to promote *fgf* expression, as depicted in Fig. 4A.

### 6. FGF Signaling is Necessary for the Exclusion of SoxB1 Expression from the Mesoderm, but not from the Early Organizer

For several of the factors that promote organizer formation but are repressed by Sox3, including Wnt and Nodal signaling, we have found that the *soxB1s* are reciprocally repressed by those factors (see diagram Fig. 4A). Since the regions where *soxB1* expression is lost at early stages coincide with the regions where the *fgf* genes are expressed, we asked whether a similar mutual inhibition between the SoxB1s and Fgf signaling might occur.

Expression of *sox3* is activated by Fgf signaling in several contexts, including its expression in the neural ectoderm, which is almost entirely lost when Fgf signaling is inhibited and is expanded throughout the ectoderm when Fgf signaling is activated [7], [20]. However, we have shown that Fgf signaling is not necessary for *sox3* expression prior to 50% epiboly in zebrafish [7] and the effect of altering Fgf signaling upon the exclusion of *sox3* expression from the mesoderm and organizer has not been analysed. We first analysed in greater detail when the expression of *sox3* and the other *soxB1* genes becomes dependent upon Fgf signaling. We found that Fgf signaling only becomes necessary for *sox3* expression at 60–70% epiboly (7–8 hpf) (See Fig. S10 in the supplementary material) after which time SU5402 caused strong inhibition of the expression of *sox3* (by 8 hpf, 70% epiboly). *sox19a* was uninhibited by SU5402 at all stages analysed, despite being expressed at significant levels at 70% epiboly. Expression of *sox19b* was also not inhbited by SU5402, although its expression was very weak by 70% epiboly, when *sox3* expression was first affected (See Fig. S10 in the supplementary material).

**Figure 6:**
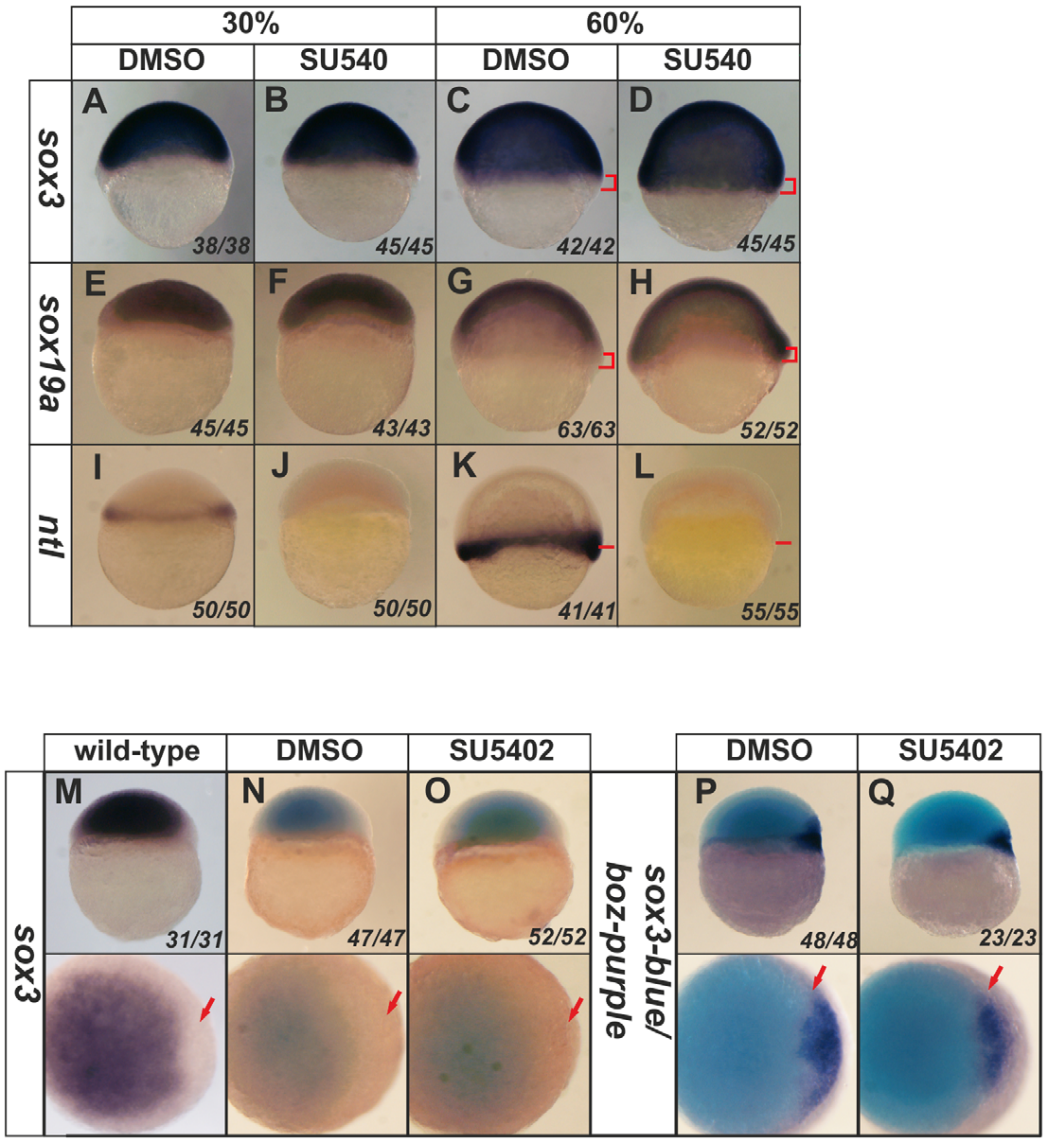
Inhibition of Fgf signaling causes expansion of *sox3* and *sox19a* expression into the vegetal margin. *In situ* hybridization for *sox3* (**A–D**), *sox19a* (**E–H**) or *ntl* (**I**–**L**). Expression of *sox3* and *sox19a* (dark purple) is seen throughout the entire animal hemisphere at the 30% epiboly stage, when only a very thin band of *ntl* expression was seen around the vegental margin (**I**). Although expression of *ntl* was completely lost following treatment with SU5402 (but not in the DMSO control) (**I**,**J**), it was not possible to detect any change in expression of *sox3* or *sox19a* (**A**,**B**,**E**,**F**). However, treatment with SU5402 also resulted in loss of expression of *ntl* by the 60% epiboly stage (**K**,**L**) (position of vegetal margin identified with red bars), which was concomitant with expansion of the expression of *sox3* and *sox19a* towards the vegetal margin (**D**,**H**) (region of expansion of expression shown with red brackets in panels C,D,G,H). Close analysis of the dorsal organizer region revealed that, as in untreated embryos (**M**), *sox3* expression is absent from the region of the organizer. Treatment with SU5402 (or DMSO as a control) did not alter the exclusion of sox3 expression from this region (**N**,**O**). The expression of *boz* (dark purple) also remained unaltered with a gap in between the domains of *sox3* and *boz* expression maintained (**P**,**Q**; arrow). All treatments were started at 3 hpf (the time that zygotic expression begins). All panels are lateral views, dorsal to the right except lower panels of **M**–**Q**, which are animal pole views. The proportion of embryos exhibiting these phenotypes is shown at the bottom right of each panel.

Closer analysis of SU5402-treated embryos at 60% epiboly revealed that, rather than losing expression, the domain of *sox3* and *sox19a* expression was expanded towards the vegetal margin (See Fig. S10A,B in the supplementary material; Fig. 6A–H), which was coincident with loss of expression of the mesoderm marker, *ntl* (Fig. 6A–H) (as described previously by Rodaway et al. (1999) [21] using a dominant-negative FGF). We also noted that inhibition of Fgf signaling resulted in stronger expression of *sox3* and *sox19a* (but not *sox19b)* throughout the embryo at 30% epiboly (Fig. 6A,C, Fig. S10 in the supplementary material). *sox19b* expression was already weak by this stage so it was not possible to be certain if a similar expansion occurred.

Since Fgf signaling appears to be necessary for the exclusion of *sox3* expression from the mesendoderm, we asked whether this was also true for its exclusion from the organizer. At 4.5 hpf, the stage when the organizer first appears as a *boz*-expressing dorsal domain, SU5402 had no effect upon the exclusion of *sox3* expression from this region (Fig. 6M–O). Given that any expansion of Sox3 would be expected to repress expression of *boz*, this result is consistent with the observation that *boz* expression is also unaffected by inhibition of Fgf signaling (Fig. 6P,Q).

Since Fgf signaling appeared to be necessary to exclude *sox3* expression from the mesendoderm but not the early organizer, we next asked if Fgf signaling was sufficient to inhibit *sox3* expression. At no stage was injection of RNA encoding Fgf3 sufficient to cause any detectable inhibition of *sox3* expression (See Fig. S11 in the supplementary material) despite being able to activate *ntl* ectopically (data not shown). Fgf signaling is therefore necessary but not sufficient for inhibition of *sox3* expression in the prospective mesoderm, but does not appear to play a role in the exclusion of *sox3* expression from the early organizer.

## Discussion

### The Role of Fgf Signaling and the SoxB1 Factors in Organizer Formation

Fgf signaling is one of the most widely functioning intercellular signaling pathways in vertebrate development. Its earliest described function is in the formation of the mesoderm and organizer. Analysis of the role of Fgf signaling in early zebrafish development implies that it might be an essential component of the signals that promote organizer formation [17], [14], [3]. In contrast, we have shown that Sox3 and other SoxB1 factors that are expressed throughout the epiblast prior to organizer formation play an opposing role in which their presence inhibits organizer formation [9].

In this study, we have shown that Fgf signaling is independently required for the expression of both *gsc* and *chd* and for the exclusion of *SoxB1* expression from the mesoderm but not from the organizer. The inhibition of *fgf3* and *fgf8* expression by Sox3 identifies yet more pro-organizer factors that are repressed by Sox3, reinforcing its role as a master repressor of the signaling that promotes organizer formation. Rather than inhibiting the expression of one key factor that is required to trigger organizer formation in the way that many regulatory networks appear to be structured, Sox3 is an inhibitor of the signaling pathways and of the target genes of those pathways. Indeed, it also represses the expression of several markers of mesoderm development in zebrafish (data not shown) and in Xenopus [22], [23]. Thus, the expression of *sox3* throughout the prospective ectoderm acts to protect ectodermal fate, limiting the expansion of the mesoderm and organizer to their very restricted vegetal domains. Since simply inhibiting the activity of the SoxB1 factors is sufficient to induce ectopic expression of all the markers studied, including the *fgfs*, it seems that this repression is one of the most important constraints on where the organizer forms. When the repression is removed, the entire process is activated where it would not normally occur.

It is noteworthy that overexpression of Fgfs can induce widespread expression of both *gsc* and *chd*, but does not cause a decrease in expression of *sox3*. This reveals that organizer genes can be expressed where there are significant levels of *soxB1* gene expression. Together, these data support the model shown in Fig. 4A, in which Fgf signaling is necessary for organizer formation downstream of Sqt signaling and, along with the other organizer promoting genes, *fgf* expression is repressed by Sox3. However, although Fgf signaling does not reciprocally repress *sox3* expression, it can override repression by the SoxB1 factors when signaling levels are increased. This implies that the precise domain of organizer gene expression results from the balance between the competing activating forces of Fgf signaling and the repressive actions of the SoxB1 factors. Endogenous Fgf signaling levels are insufficient to induce organizer gene expression where Sox3 is expressed, such that it is Sox3 that defines the limit of their expression. However, even when SoxB1 factor activity is blocked using a dominant negative construct, Fgf signaling is still needed for the resulting ectopic expression of organizer markers. Hence, Fgf signaling is also needed independently to promote organizer gene expression.

It is interesting to note that Fgf signaling can independently promote strong ectopic expression of *gsc* and *chd* but does not cause full axis duplication ([24] and our own unpublished observations), implying that it does not generate a true ectopic organizer. Since Fgf signaling does not induce *boz* or *sqt* expression, this supports a role for Boz and/or Sqt in promoting other aspects of organizer formation in addition to simply inducing expression of the *fgfs* which are necessary to maintain continued organizer development.

Overall, our data lead to a model for organizer formation in which widespread expression of the SoxB1 factors restricts organizer formation by inhibiting Fgf, Nodal and Wnt signaling, as well as independently repressing the targets of those signaling pathways. The organizer therefore forms only where Nodal-induced Fgf signaling overlaps with Wnt signaling and the SoxB1 proteins are absent. Since SoxB1 factors are initially present throughout the epiblast, this would preclude organizer formation at an earlier stage. *SoxB1* expression is only lost at about the time that the organizer marker, *boz*, is first expressed. Since Boz and Sox3 exhibit mutual repression, it is not clear whether the appearance of *boz* is causative of the loss of *sox3* expression, or if the two events are independent consequences of other upstream signals. The timing of *fgf* expression, a little later than these events, suggests that initiation of *fgf* expression is dependent on the signals that promote their expression rather than de-repression due to the loss of *soxB1* expression from the region of the organizer.

A similar role for Fgfs in organizer formation has been suggested from studies over many years in organisms as diverse as *Xenopus*, chick and mouse (reviewed in Bottcher and Niehrs 2005 [25]). In all three organisms, Fgf signaling is a major component necessary for mesoderm formation. In addition to this, interfering with Fgf signaling disrupts dorsoventral patterning and the morphogenetic movements that occur during gastrulation [25]. However, evidence in support of a direct role for Fgf signaling in organizer formation has only recently been reported [3].

Although the role of Sox3 has not been studied with respect to *fgf* expression in these other animal models, the expression of *sox3* in chick and in *Xenopus* is consistent with a similar role (such detailed expression data is not available for mouse). In both *Xenopus* and chick embryos, early expression of *sox3* is throughout the epiblast but loss of expression precedes gastrulation in the region equivalent to the zebrafish organizer [26], [27].

### Context Dependent Interaction between the SoxB1 Family and Fgf Signals

The expression of *soxB1* genes, including *sox3*, has been shown to be dependent upon Fgf signaling in several regions of neural epithelium, such as in the placodes and later otic neural epithelium [4], [6]. Indeed, it is clear from our studies that expression of *sox3* in the neural ectoderm of the CNS becomes dependent upon Fgf signaling between 7 and 8 hpf in zebrafish. However, expression of *sox3* at earlier stages does not require Fgf signaling [7], when Fgfs instead play a role in repressing *soxB1* expression in the mesendoderm. Since over expression of Fgfs alone is insufficient to expand the inhibition of *sox3* expression beyond the normal domain of the mesendoderm, it seems that the mechanism for this requires additional factors that are restricted to the marginal region of the embryo.

It is not yet known why *sox3* expression might be dependent upon Fgf signaling at later stages, but is repressed or insensitive to it at earlier stages. Our earlier study showed that *sox3* expression only became dependent upon Fgf signaling at the same time that it became sensitive to inhibition by Bmp signaling [7]. Fgf is known to directly repress Bmp signaling by triggering inactivating phosphorylation of Smad proteins, the intracellular effectors of Bmp signaling. Thus, it seems that a change in the molecular machinery of Bmp signaling at this early stage of development fundamentally alters the state of the embryonic cells. Prior to about 60% epiboly all embryonic cells express *sox3*, and this expression is insensitive to Bmp signaling [7]. After this stage, changes downstream of Bmp receptor activation cause repression of *sox3* expression, but Fgf signaling then protects *sox3* from this repression in dorsal and marginal regions (as shown by the fact that sox3 expression is broadly lost when Fgf signaling is inhibited) [7].

### Context Dependent Actions of Sox3

Although every gene target we have analyzed in the mesoderm and organizer is repressed by Sox3, this is not true in other embryonic contexts. Once neural induction occurs, a process in which Sox3 also plays an active role [28], [29], Sox3 acts largely as an activator of transcription. In this context it appears to promote the neural stem cell state and inhibit differentiation primarily through maintaining the expression of stem cell related genes [30]. However, some data suggest that SoxB1 factors also directly repress gene expression, even at this stage of development [31], [32]. This therefore raises the question of how these alternative modes of action, repressing some gene targets and activating others, might be achieved. One mechanism by which these actions could be regulated is by the cell-context availability of cofactors. A variety of cofactors are known that can alter the activity of transcription factors between activator and repressor functions. One class of protein co-repressors is the Groucho family. For example, interaction between the Sox-like HMG factor, Tcf, and Grouchos leads it to repress its targets, while binding to ß-catenin releases Tcf from this interaction such that it activates its target genes [33], [34], [35]. Sox3 also interacts with members of the Groucho family (our unpublished data) providing one mechanism by which its activity may be switched. Whichever cofactor type it uses, the data we have presented in this study may be in part explained by a change in the presence of cofactors at different stages of development or in different regions of the embryo, with corepressors present during organizer formation, and co-activators present during neural induction. However, the action of the SoxB1 factors also appears to be controlled by the gene target with which they associate. This is particularly evident from the effects we see when overexpressing the HMG-enR or HMG-VP16 constructs. The HMG-enR construct represses the genes that are repressed by the WT Sox3, but the HMG-VP16 construct has no effect upon these genes. Conversely, the HMG-VP16 construct activates the genes activated by Sox3 at later stages, but the HMG-enR construct has no effect upon these (our unpublished data). Thus, whether these constructs are able to activate or repress a gene is determined, at least in part, by the target gene in question. We do not, at this stage, know what the mechanism of this effect may be, but we would suggest that it is mediated via the recruitment of key, DNA sequence-dependent co-activators or co-repressors or even at the level of DNA/chromatin structure.

It is noteworthy that, while in many previous studies enR and VP16 fusion constructs have been able to exert their reciprocal effects on the same genes, a similar effect to that described here (in which genes were only affected by either the enR or the VP16 construct) was seen for the transcription factor, FoxD5, in *Xenopus* [36]. This implies that the sequence dependence of these opposite transcriptional activities may be a more general phenomenon, not restricted to the SoxB1 proteins.

Overall, this study adds new depth to our understanding of the complex interaction of signals and transcription factors that ensure that one of the earliest and most fundamental patterning events of vertebrate development occurs at the correct time and in the correct place.

## Materials and Methods

### RNA Injection

Embryos obtained from natural crosses were maintained at 28.5°C and staged according to hours postfertilization (hpf) and morphological criteria [37]. All capped sense mRNAs for microinjection were synthesized from linearized cDNA template and purified using the mMessage-Machine Kit (Ambion, Life technologies, Paisley, UK). Embryos were injected with 50–200 pg of RNA at the 1–2 cell stage. The dominant negative form of FGFR-1, XFD, and D50 as a negative control were a kind gift from Professor Enrique Amaya; *mkp3* was a kind gift from Dr. Igor B. Dawid.

### Morpholino Injection

Antisense morpholino oligonucleotides (MOs) were designed to target the 5′ region of *sqt* [3], *fgf3* [38] and *fgf8* [39] from Gene Tools (Philomath, OR, USA). Embryos were injected with 5–10 ng MO in 0.5 nl water at the 1–2 cell stage.

### SU5402 Treatment

3-[(3-(2-carboxvethyl)-4-methylpyrrol-2-yl) methylene]-2-indolinone (SU5402) (Calbiochem, Nottingham, UK) was used at a final concentration of 84 µM in fish water containing methyl blue. Embryos were treated with SU5402 from 2.5 hpf before (zygotic gene expression was initiated) until the required stage of collection. As a negative control, embryos were treated with an equivalent concentration of DMSO.

### Whole-mount *in situ* Hybridisation

Whole-mount *in situ* hybridisation was carried out as previously described [40] using labelled riboprobes. Labelled riboprobes were transcribed from linearised templates using T3, T7 or SP6 RNA polymerase (Promega, Madison WI, USA) in the presence of DIG-labelled or fluorescein-labelled nucleotides (Roche, Basel, Switzerland). Antibodies were detected using BM purple, BCIP (Roche, Basel, Switzerland) or Fast-Red (Sigma, St-Louis, Missouri, USA). For double *in situ* hybridisation, following hybridisation with a combination of two riboprobes labelled with DIG and fluorescein, sequential detection was carried out with AP-conjugated antibodies. The enzymic activity was blocked between detection of the DIG and the fluorescein 0.1 M glycine-hydrochloride, pH 2.2, 0.1% Tween 20. The two rounds of antibody/colour detection used combinations of BM purple with either or BCIP alone (Roche, Basel, Switzerland) or Fast-Red (Sigma, St-Louis, Missouri, USA).

### ChIP-PCR

Embryos at the 1–2 cell stage were injected with RNA and collected at 4.5 hpf. Dechorionated embryos were fixed in 1.85% formaldehyde. After quenching with 2.5 M glycine, embryos were washed and then lysed in 10 mM Tris-HCl pH 7.4, 10 mM NaCl, 0.5% NP40. Nuclei were pelleted in a microcentrifuge at 1000 *g* for 5 minutes and resuspended in 50 mM Tris-HCl pH 7.4, 10 mM EDTA, 1% SDS. Two volumes of IP dilution buffer (16.7 mM Tris-HCl pH 7.4, 167 mM NaCl, 1.2 mM EDTA, 1.1% Triton X-100, 0.01% SDS) were added and samples sonicated and then centrifuged at 14,000 *g* for 10 minutes. Supernatant was incubated with HA beads (HA agarose, A-2095, Sigma) at 4°C overnight. Beads were washed eight times with wash buffer (50 mM Hepes pH 7.6, 1 mM EDTA, 0.7% sodium deoxycholate, 1% NP40, 0.5 M LiCl) and once with 1xTBS [50 mM Tris-HCl (pH 7.4), 150 mM NaCl] and the DNA-protein complex was eluted in 50 mM Tris-HCl pH 8, 10 mM EDTA, 1% SDS at 65°C overnight. After treatment with proteinase K at 55°C for 2 hours, DNA was precipitated in ethanol. Real-time PCR was carried out using MX3005P MX-PRO (Stratagene, cedar creek, Texas, USA) and Brilliant SYBR Green Master Mix Kit (Stratagene, cedar creek, Texas, USA) with the following primers (5′ to 3′): *tubb5*-F CCCAATTTTAAAACACGCCTA, *tubb5*-R CGGATGAGG ACGATTTAACC, *fgf3*-1F CCGACATGCATCTTCTCTCA, *fgf3*-1R CCCACGAGGTTTTCAATAGC, *fgf3*-2F CCGAAGAGATTTTGGTGCTT, *fgf3*-2R CAGGCCCTCAGATCACTAGC, *fgf3*-3F TTTGCGCTAGTGATCTGAGG, *fgf3*-3R TCAAACCAACCTGAGGTAATGA, *fgf3*-4F TTGGGAGGACAGTGGATTTC, *fgf3*-4R AATCGCAAGATTCGGACAAT, *fgf3*-5F GGATAGGGCTTTCCTTTTGG, *fgf3*-5R CCTGCATGGAGCTGTGTAAA.

### Bioinformatic Analyses

Vertebrate FGF3 orthologues were identified using the ENSEMBL database (www.ensembl.org). Nucleotide sequences containing the coding and 5′ regions for each gene were downloaded and conserved regions identified using PipMaker [13]. Highly conserved regions upstream of the FGF3 coding region were then aligned using ClustalW2 (www.ebi.ac.uk).

## Supporting Information

Figure S1 **Endogenous expression of (A)** ***fgf3*** **and (B)** ***fgf8*** **first could first be detected in the organizer region at 4.5 hpf during early zebrafish development.** Lateral view and dorsal is to the right in upper panels, viewed from animal pole in lower panels.
(TIF)

Figure S2 **dnSox3 and wild-type Sox3 counteract eachother’s effects on** ***fgf8*** **expression.** Injection of RNA encoding wild-type Sox3, Sox19a or Sox19b at the 1–2 cell stage caused disruption of endogenous *fgf8* expression (gaps in expression, arrow heads in upper panels, brackets in lower panels) (**Aa**–**d**). Injected of *dnSox3* RNA at the 1–2 cell stage caused ectopic expression (arrow) and expansion of the endogenous domain of *fgf8* expression (**Ae**), but this was rescued by co-injecting RNA encoding wild-type Sox3, Sox19a or Sox19b with the majority of embryos reverting to fgf8 expression equivalent to that seen in uninjected embryos (**Af–h**). (**B**) Graphical representation of the numbers of embryos affected in these experiments. Lateral view and dorsal is to the right in upper panels, viewed from animal pole in lower panels. The proportion of embryos exhibiting these phenotypes is shown at the bottom right of each panel.
(TIF)

Figure S3 **Aligment of genomic regions upstream of fgf3 across diverse species.** Left panel shows PIP plot of the region upstream of *fgf3*, distances marked as kb (k). *fgf3* gene shown as ‘underlay’ in yellow with coding regions in blue, UTRs in orange and introns in yellow. Green bars show regions with >50% identity to the zebrafish sequence, red bars indicate regions with >75% identity to the zebrafish sequence. Right panel is a detailed PIP plot showing *fgf3* gene in yellow with exons in blue. Top line shows repeat elements as arrow heads and open boxes, exons are numbered boxes and the orientation of the gene by an arrow. Dots represent regions showing similarity to zebrafish, the height of the dots within each bar indicate the % nucleotide identity. Numbering relates to the zebrafish genome relative to the *fgf3* transcription start site at position 28694.
(JPG)

Figure S4 **Clustal alignments of genomic regions upstream of fgf3 across diverse species.** (A–D as described in Fig. 2). Sox binding consensus sequences in gray boxes. Stars show bases entirely conserved in species shown. Numbering relates to the zebrafish genome relative to the *fgf3* transcription start site at position 28694.
(DOCX)

Figure S5 **Single morpholinos targeting** ***fgf3*** **or** ***fgf8*** **have limited inhibitory effects on the expression of** ***chd*** **and** ***gsc*** **.** Injection of an *fgf3*MO (5 ng) at the 1–2 cell stage caused a substantial, but incomplete, reduction in the domain of expression of *chd* and *gsc* at 4.5 hpf (**A–D**). Injection of an *fgf8*MO (5 ng) at the 1–2 cell stage caused a significant, but lesser, inhibition of *chd* and *gsc* expression (**E,F**). Lateral view and dorsal is to the right in upper panels, viewed from animal pole in lower panels. The proportion of embryos exhibiting these phenotypes is shown at the bottom right of each panel.
(TIF)

Figure S6 **When** ***Sox3*** **is coinjected with** ***FGF3*** **or** ***Gsc*** **, gaps in ectopically-induced** ***Chd*** **expression coincide with the region of highest sox3 expression.** Embryos were injected with 50 pg of *sox3* plus 50 pg of either *fgf3* RNA (A,B) or *gsc* RNA (C,D) and analysed for *chd* expression (blue/purple) at 4.5 hpf. Sox3 and gsc protein was detected by virtue of the HA tags they carried, using a brown peroxidase substrate. In each case the predominant region of sox3 overexpression corresponded with a gap in the region of ectopically-induced *chd* expression although there was often some overlap where deeper *chd*-expressing cells appeared to be overlaid by weaker sox3 overexpressing cells nearer the surface. Viewed from animal pole.
(TIF)

Figure S7 **Sox3 overexpression is able to inhibit the ectopic expression of** ***chd*** **induced by a range and combination of upstream factors.** Embryos were injected with 50 pg of various RNAs (indicated above each panel) alone or combination and analysed for *chd* expression at 4.5 hpf (**A**). Injection of *boz*, *fgf3,* or *boz* combined with *fgf3,* caused expansion of *chd* expression into the animal hemisphere of embryos (**Ab–d**). Injection of the additional up-stream factors, *S37A* (constitutive active *ß-catenin*) and *sqt* strongly induced expansion of *chd* throughout the entire animal hemisphere (**Ae**). However, co-injected with *sox3* (**B**) led to reduced expansion or negative patches in the expansion of *chd* expression no matter which other factors were injected. Although the combination of all factors still gave strongest extopic expression, co-injection of *sox3* was still able to generate *chd* negative patches (**Be**). Lateral view and dorsal is to the right in upper panels, viewed from animal pole in lower panels. The proportion of embryos exhibiting these phenotypes is shown at the bottom right of each panel.
(TIF)

Figure S8 **Sox3 overexpression is able to inhibit the ectopic expression of** ***gsc*** **induced by a range and combination of upstream factors.** Embryos were injected with 50 pg of various RNAs (indicated above each panel) alone or combination and analysed for *gsc* (**A**) expression at 4.5 hpf. Injection of *boz*, *fgf3,* or *boz* combined with *fgf3,* caused expansion of *gsc* expression into the animal hemisphere of embryos (**Ab–d**). Injection of the additional up-stream factors, *S37A* (constitutive active *β-catenin*) and *sqt* strongly induced expansion of *gsc* throughout the entire animal hemisphere (**Ae**). However, co-injection with *sox3* (**B**) reduced the expansion of expression of *gsc* expression or generated negative patches in the expansion of *gsc* expression no matter which other factors were injected. Although the combination of all factors still gave strongest ectopic expression, co-injection of *sox3* was still able to generate *gsc* negative patches (**Be**). Lateral view and dorsal is to the right in upper panels, viewed from animal pole in lower panels. The proportion of embryos exhibiting these phenotypes is shown at the bottom right of each panel.
(TIF)

Figure S9 **Overexpression of Fgf3 can rescue the loss of** ***gsc*** **and** ***chd*** **expression following knockdown of Sqt.** Embryos were injected with *fgf3* RNA (50 pg) or *sqt*MO (10 ng) or a combination of both and analysed for *chd* and *gsc* expression at 4.5 hpf. Endogenous expression of *chd* and *gsc* (**A,E**) was expanded into the animal hemisphere of embryos by injection of *fgf3* at 1–2 cell stage (**B,F**). Injection of *sqt*MO at the 1–2 cell stage caused partial repression of *chd* expression (**C**) and complete loss of *gsc* expression (**G**). These inhibitory effects of the *sqt*MO could be rescued by co-injection of *fgf3* RNA (**D**,**H**). Lateral view and dorsal is to the right in upper panels, viewed from animal pole in lower panels. The proportion of embryos exhibiting these phenotypes is shown at the bottom right of each panel.
(TIF)

Figure S10 **Expression of** ***sox3,*** **but not** ***sox19a*** **or** ***sox19b,*** **becomes Fgf-dependent between 60 and 70% epiboly.** Embryos were treated with 84 µM SU5402 or DMSO alone from 3 hpf (the time that zygotic expression begins) and the expression of *sox3* (**A**) *sox19a* (**B**) or *sox19b* (**C**) genes was analyzed at 30%, 60% and 70% epiboly. The expression patterns of *sox3/19a/19b* in embryos treated with SU5402 were the same as DMSO treated embryos at both 30% and 60% epiboly stage. At 70% epiboly, only *sox3* expression was affected by SU5402, when it was strongly inhibited in embryos treated with SU5402. Lateral view and dorsal is to the right in upper panels, viewed from animal pole in lower panels. The proportion of embryos exhibiting these phenotypes is shown at the bottom right of each panel.
(TIF)

Figure S11 **Over-expression of Fgf3 does not affect** ***sox3/19a/19b*** **expression.** Embryos injected with 50 pg *fgf3* mRNA at the 1–2 cell stage and the expression of *sox3* (**A**) *sox19a* (**B**) or *sox19b* (**C**) was analysed at 30%, 60% and 70% epiboly. At no stage did injection of *fgf3* RNA have any effect upon *soxB1* gene expression. Lateral view and dorsal is to the right in upper panels, viewed from animal pole in lower panels. The proportion of embryos exhibiting these phenotypes is shown at the bottom right of each panel.
(TIF)
