# Supplementary figures and images for: Formation of the Embryonic Organizer Is Restricted by the Competitive Influences of Fgf Signaling and the SoxB1 Transcription Factors
Source: PLoS One. 2013 Feb 28;8(2):e57698. doi: 10.1371/journal.pone.0057698 (PMC3585176; doi:10.1371/journal.pone.0057698)

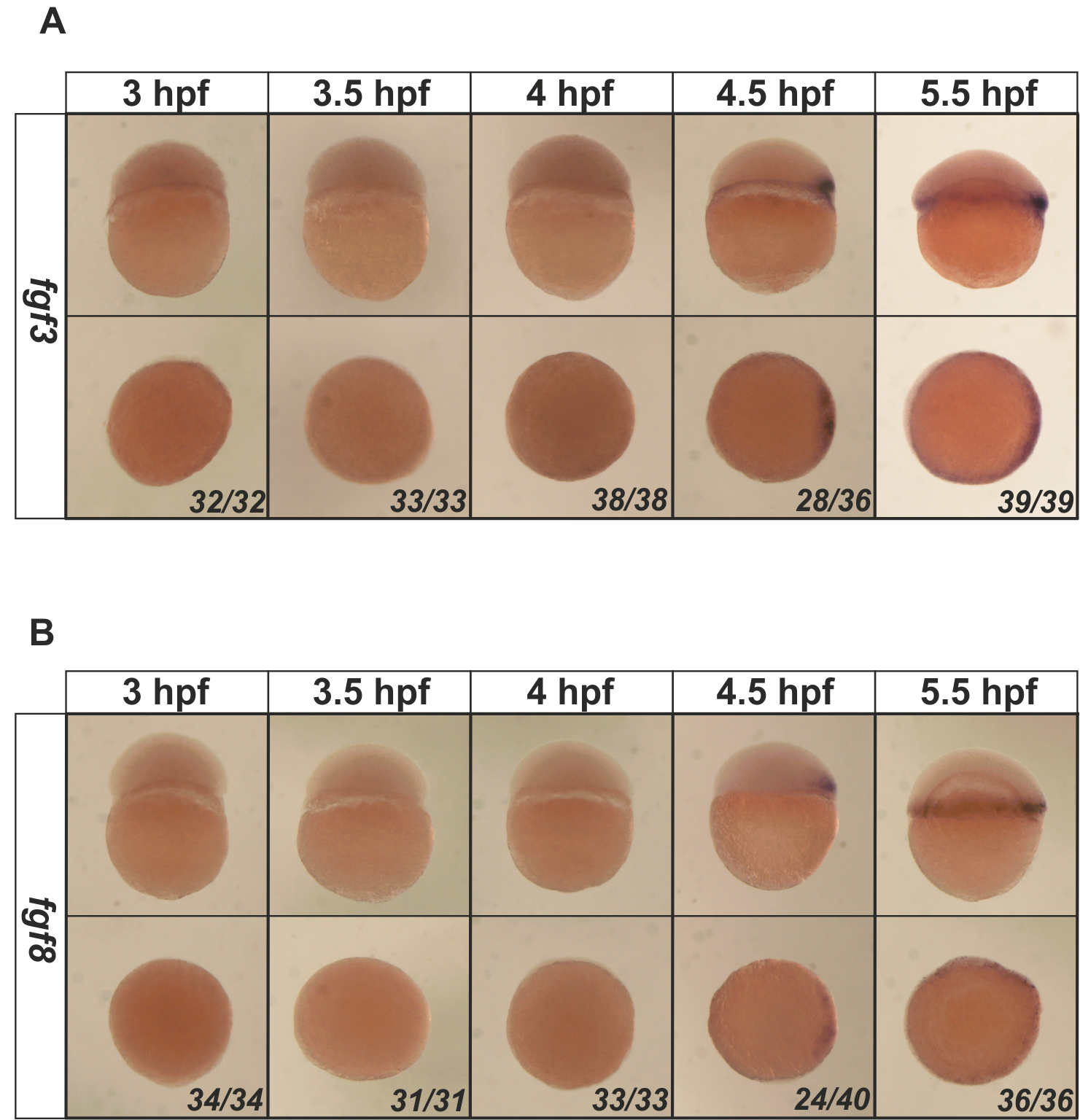

Supplement: Figure S1 — Endogenous expression of (A) fgf3 and (B) fgf8 first could first be detected in the organizer region at 4.5 hpf during early zebrafish development. Lateral view and dorsal is to the right in upper panels, viewed from animal pole in lower panels. (TIF) [file pone.0057698.s001.tif]

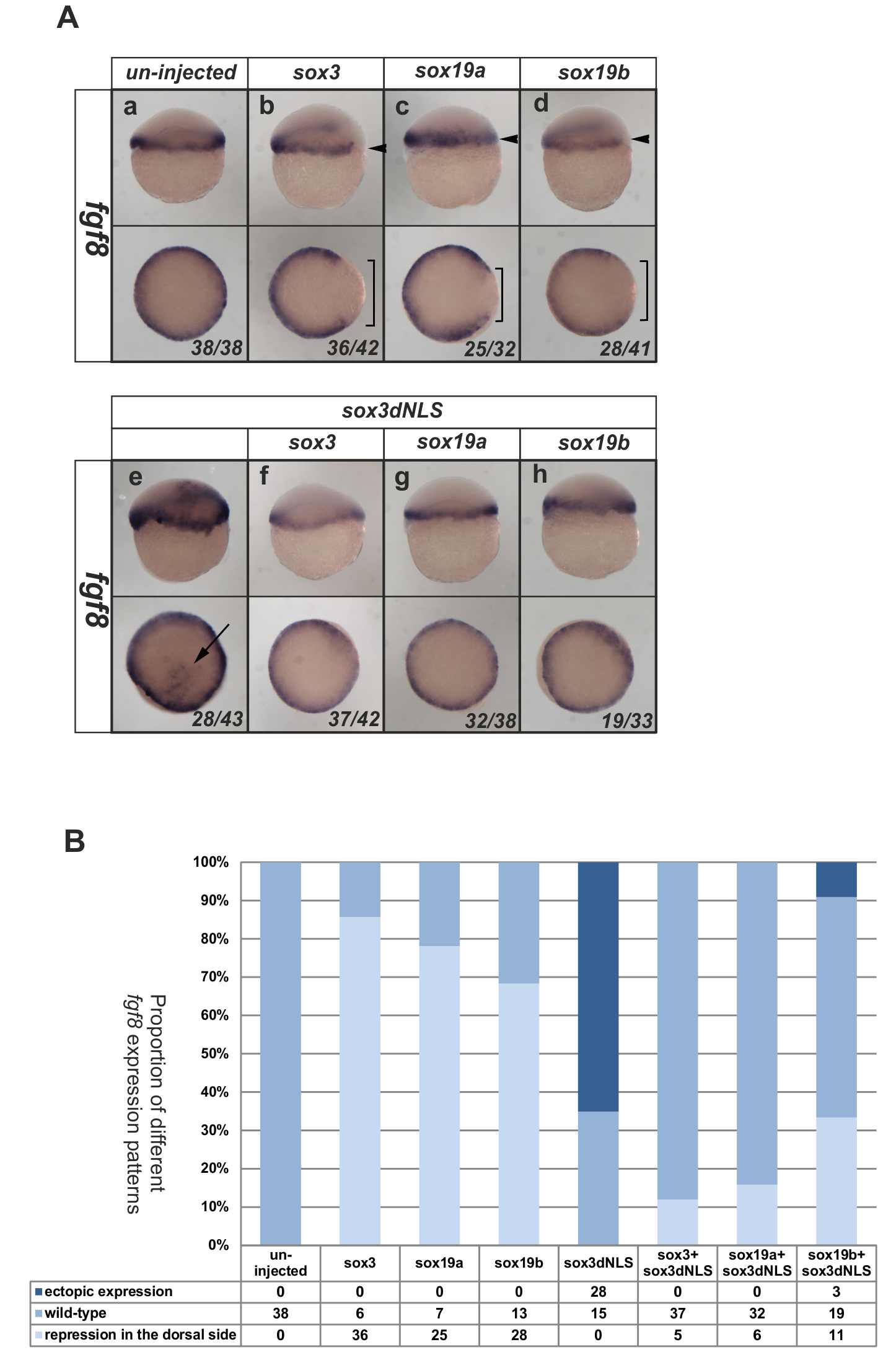

Supplement: Figure S2 — dnSox3 and wild-type Sox3 counteract eachother’s effects on fgf8 expression. Injection of RNA encoding wild-type Sox3, Sox19a or Sox19b at the 1–2 cell stage caused disruption of endogenous fgf8 expression (gaps in expression, arrow heads in upper panels, brackets in lower panels) (Aa–d). Injected of dnSox3 RNA at the 1–2 cell stage caused ectopic expression (arrow) and expansion of the endogenous domain of fgf8 expression (Ae), but this was rescued by co-injecting RNA encoding wild-type Sox3, Sox19a or Sox19b with the majority of embryos reverting to fgf8 expression equivalent to that seen in uninjected embryos (Af–h). (B) Graphical representation of the numbers of embryos affected in these experiments. Lateral view and dorsal is to the right in upper panels, viewed from animal pole in lower panels. The proportion of embryos exhibiting these phenotypes is shown at the bottom right of each panel. (TIF) [file pone.0057698.s002.tif]

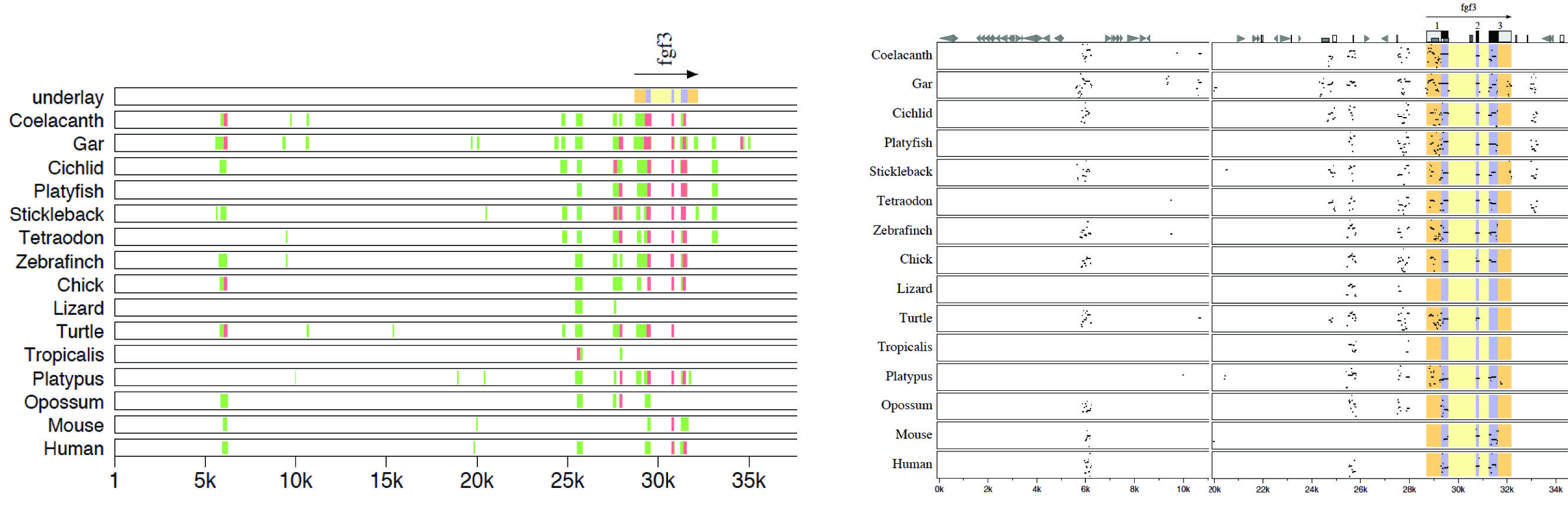

Supplement: Figure S3 — Aligment of genomic regions upstream of fgf3 across diverse species. Left panel shows PIP plot of the region upstream of fgf3, distances marked as kb (k). fgf3 gene shown as ‘underlay’ in yellow with coding regions in blue, UTRs in orange and introns in yellow. Green bars show regions with >50% identity to the zebrafish sequence, red bars indicate regions with >75% identity to the zebrafish sequence. Right panel is a detailed PIP plot showing fgf3 gene in yellow with exons in blue. Top line shows repeat elements as arrow heads and open boxes, exons are numbered boxes and the orientation of the gene by an arrow. Dots represent regions showing similarity to zebrafish, the height of the dots within each bar indicate the % nucleotide identity. Numbering relates to the zebrafish genome relative to the fgf3 transcription start site at position 28694. (JPG) [file pone.0057698.s003.jpg]

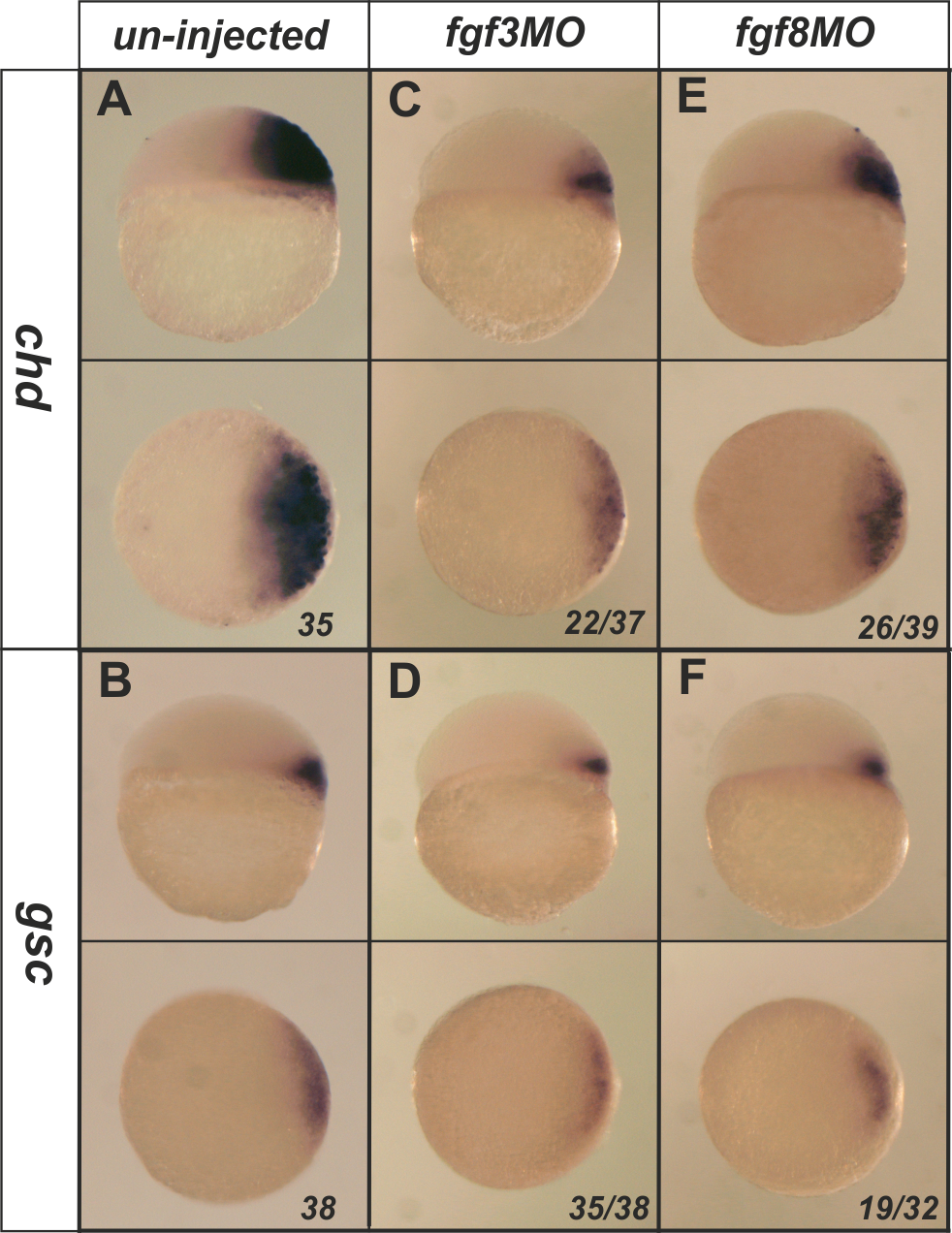

Supplement: Figure S5 — Single morpholinos targeting fgf3 or fgf8 have limited inhibitory effects on the expression of chd and gsc . Injection of an fgf3MO (5 ng) at the 1–2 cell stage caused a substantial, but incomplete, reduction in the domain of expression of chd and gsc at 4.5 hpf (A–D). Injection of an fgf8MO (5 ng) at the 1–2 cell stage caused a significant, but lesser, inhibition of chd and gsc expression (E,F). Lateral view and dorsal is to the right in upper panels, viewed from animal pole in lower panels. The proportion of embryos exhibiting these phenotypes is shown at the bottom right of each panel. (TIF) [file pone.0057698.s005.tif]

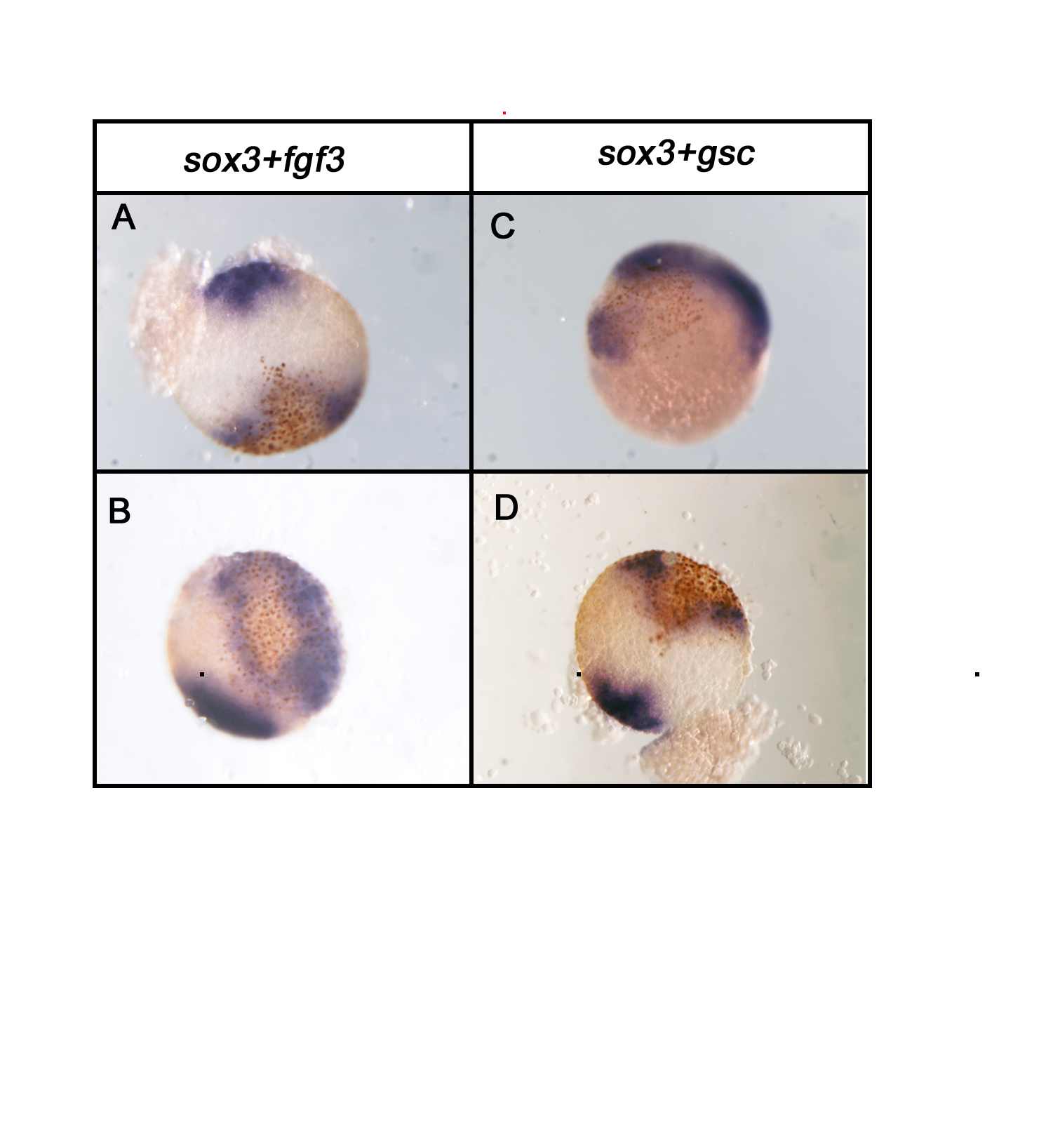

Supplement: Figure S6 — When Sox3 is coinjected with FGF3 or Gsc , gaps in ectopically-induced Chd expression coincide with the region of highest sox3 expression. Embryos were injected with 50 pg of sox3 plus 50 pg of either fgf3 RNA (A,B) or gsc RNA (C,D) and analysed for chd expression (blue/purple) at 4.5 hpf. Sox3 and gsc protein was detected by virtue of the HA tags they carried, using a brown peroxidase substrate. In each case the predominant region of sox3 overexpression corresponded with a gap in the region of ectopically-induced chd expression although there was often some overlap where deeper chd-expressing cells appeared to be overlaid by weaker sox3 overexpressing cells nearer the surface. Viewed from animal pole. (TIF) [file pone.0057698.s006.tif]

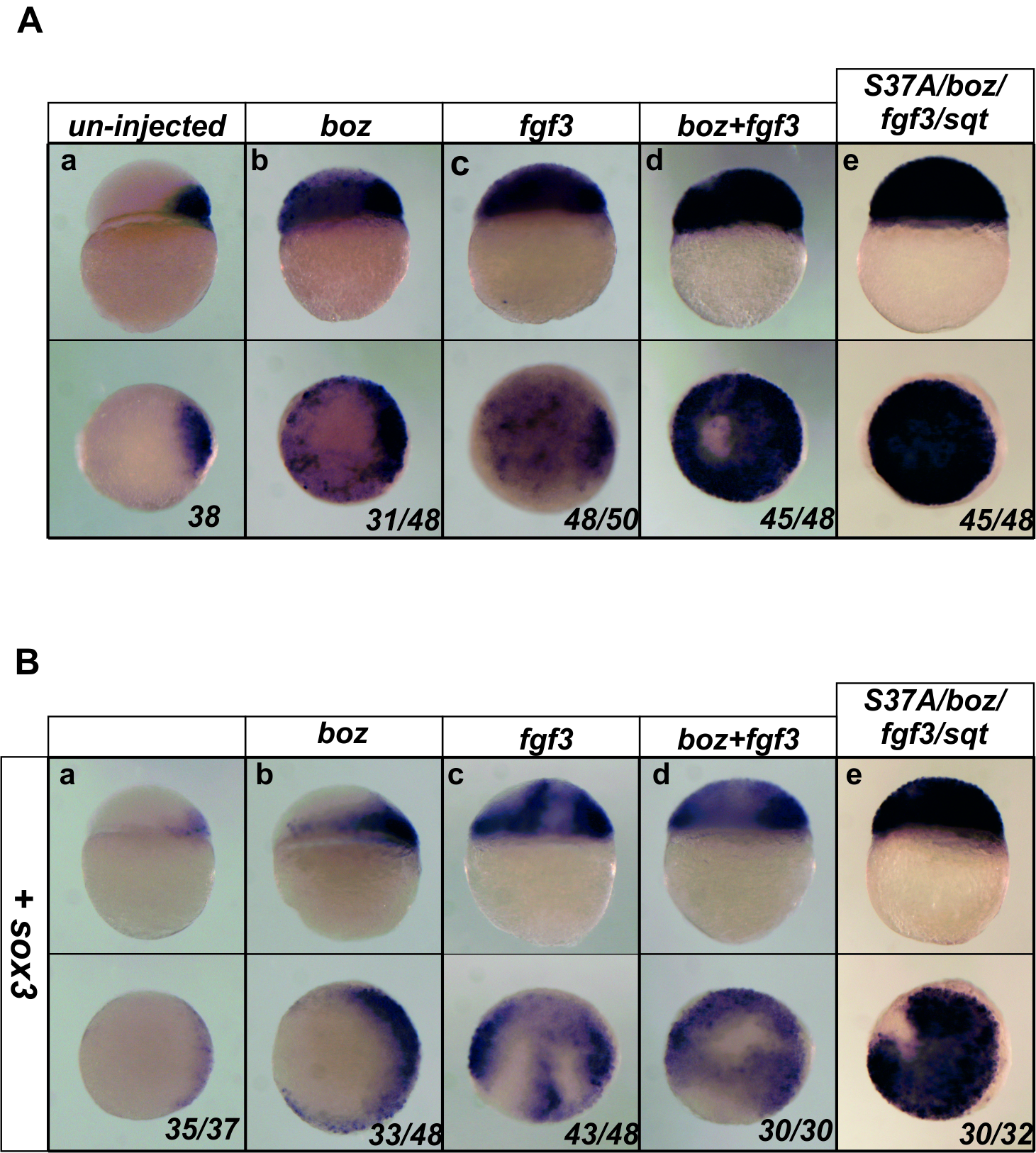

Supplement: Figure S7 — Sox3 overexpression is able to inhibit the ectopic expression of chd induced by a range and combination of upstream factors. Embryos were injected with 50 pg of various RNAs (indicated above each panel) alone or combination and analysed for chd expression at 4.5 hpf (A). Injection of boz, fgf3, or boz combined with fgf3, caused expansion of chd expression into the animal hemisphere of embryos (Ab–d). Injection of the additional up-stream factors, S37A (constitutive active ß-catenin) and sqt strongly induced expansion of chd throughout the entire animal hemisphere (Ae). However, co-injected with sox3 (B) led to reduced expansion or negative patches in the expansion of chd expression no matter which other factors were injected. Although the combination of all factors still gave strongest extopic expression, co-injection of sox3 was still able to generate chd negative patches (Be). Lateral view and dorsal is to the right in upper panels, viewed from animal pole in lower panels. The proportion of embryos exhibiting these phenotypes is shown at the bottom right of each panel. (TIF) [file pone.0057698.s007.tif]

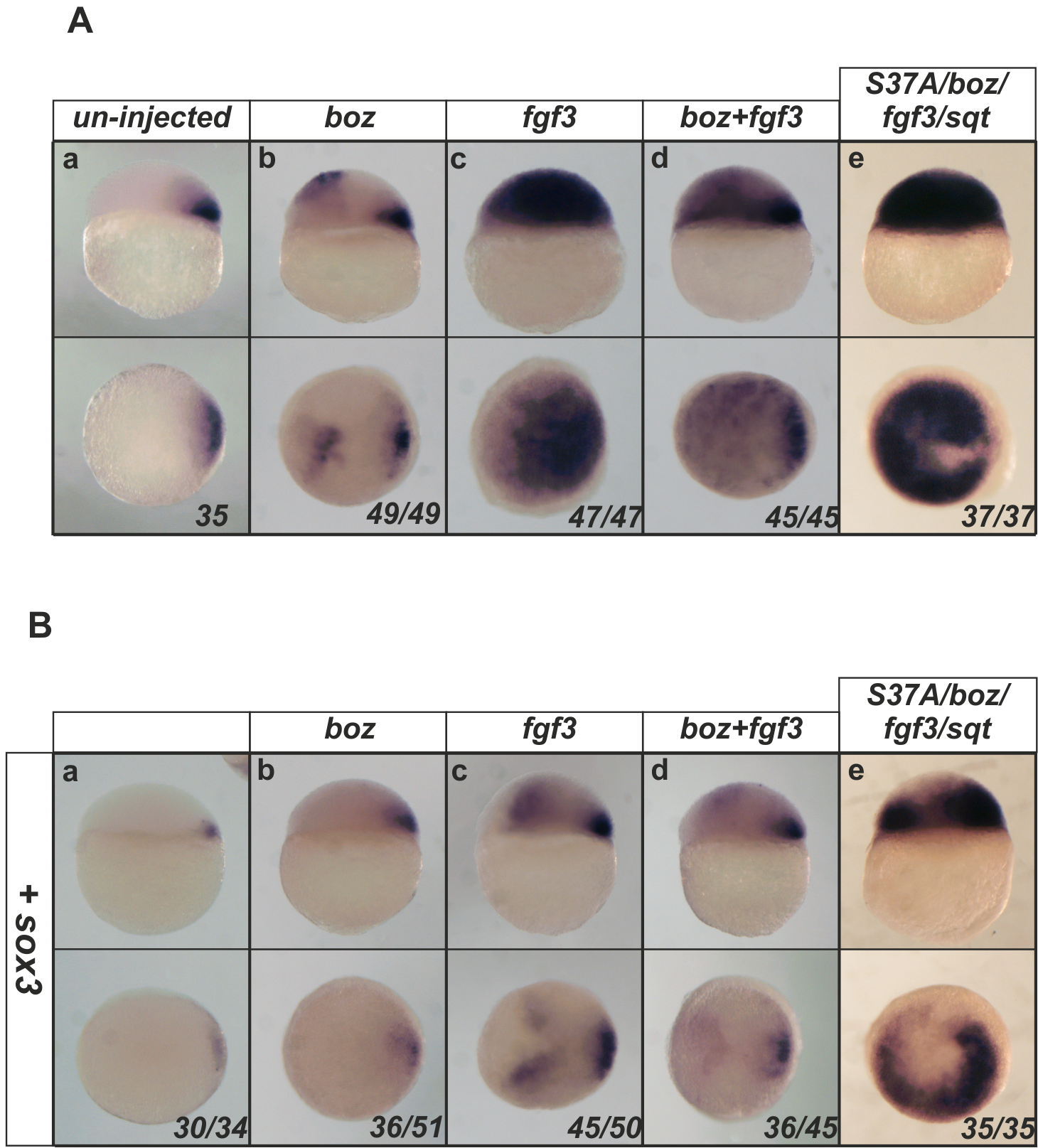

Supplement: Figure S8 — Sox3 overexpression is able to inhibit the ectopic expression of gsc induced by a range and combination of upstream factors. Embryos were injected with 50 pg of various RNAs (indicated above each panel) alone or combination and analysed for gsc (A) expression at 4.5 hpf. Injection of boz, fgf3, or boz combined with fgf3, caused expansion of gsc expression into the animal hemisphere of embryos (Ab–d). Injection of the additional up-stream factors, S37A (constitutive active β-catenin) and sqt strongly induced expansion of gsc throughout the entire animal hemisphere (Ae). However, co-injection with sox3 (B) reduced the expansion of expression of gsc expression or generated negative patches in the expansion of gsc expression no matter which other factors were injected. Although the combination of all factors still gave strongest ectopic expression, co-injection of sox3 was still able to generate gsc negative patches (Be). Lateral view and dorsal is to the right in upper panels, viewed from animal pole in lower panels. The proportion of embryos exhibiting these phenotypes is shown at the bottom right of each panel. (TIF) [file pone.0057698.s008.tif]

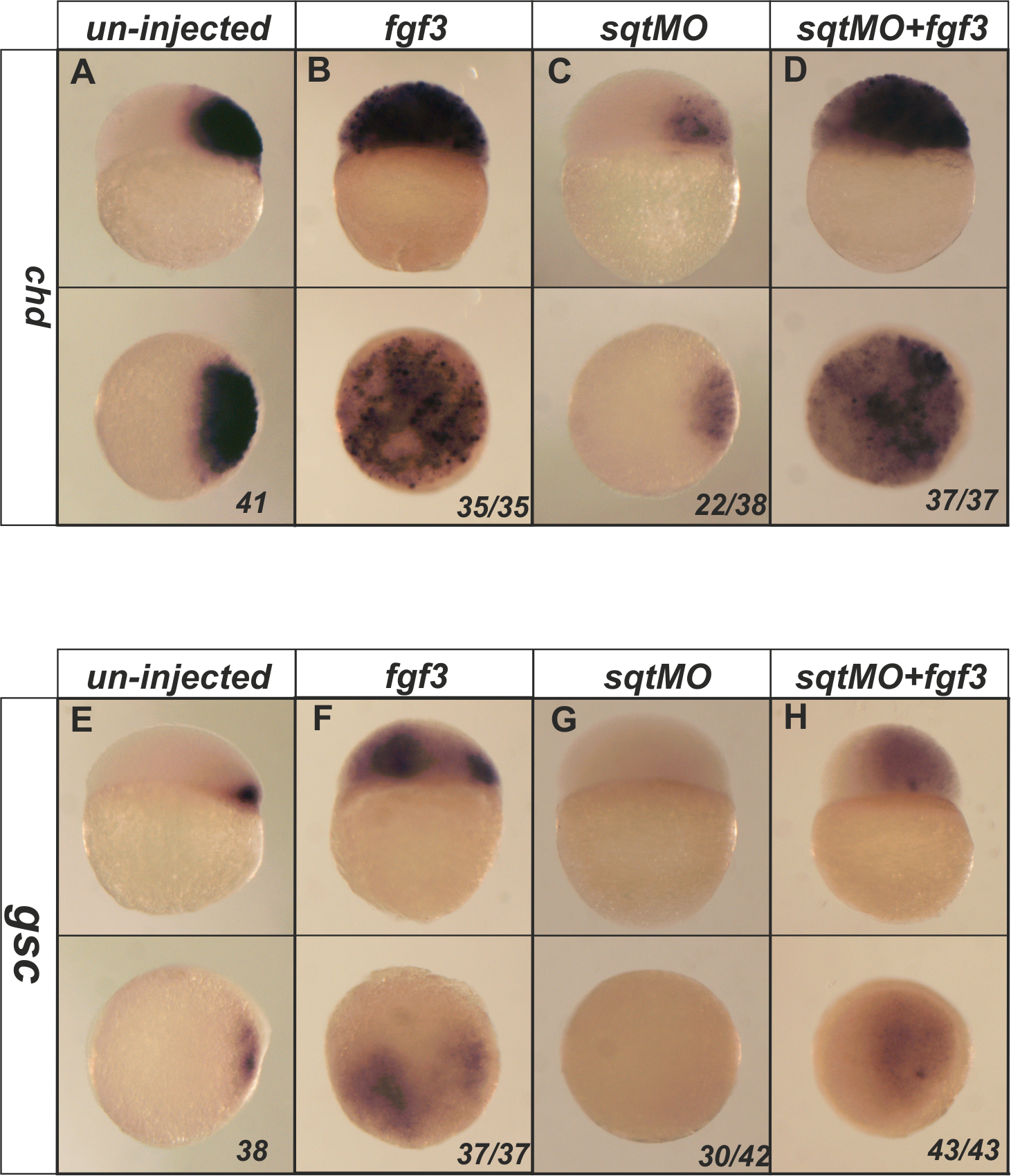

Supplement: Figure S9 — Overexpression of Fgf3 can rescue the loss of gsc and chd expression following knockdown of Sqt. Embryos were injected with fgf3 RNA (50 pg) or sqtMO (10 ng) or a combination of both and analysed for chd and gsc expression at 4.5 hpf. Endogenous expression of chd and gsc (A,E) was expanded into the animal hemisphere of embryos by injection of fgf3 at 1–2 cell stage (B,F). Injection of sqtMO at the 1–2 cell stage caused partial repression of chd expression (C) and complete loss of gsc expression (G). These inhibitory effects of the sqtMO could be rescued by co-injection of fgf3 RNA (D,H). Lateral view and dorsal is to the right in upper panels, viewed from animal pole in lower panels. The proportion of embryos exhibiting these phenotypes is shown at the bottom right of each panel. (TIF) [file pone.0057698.s009.tif]

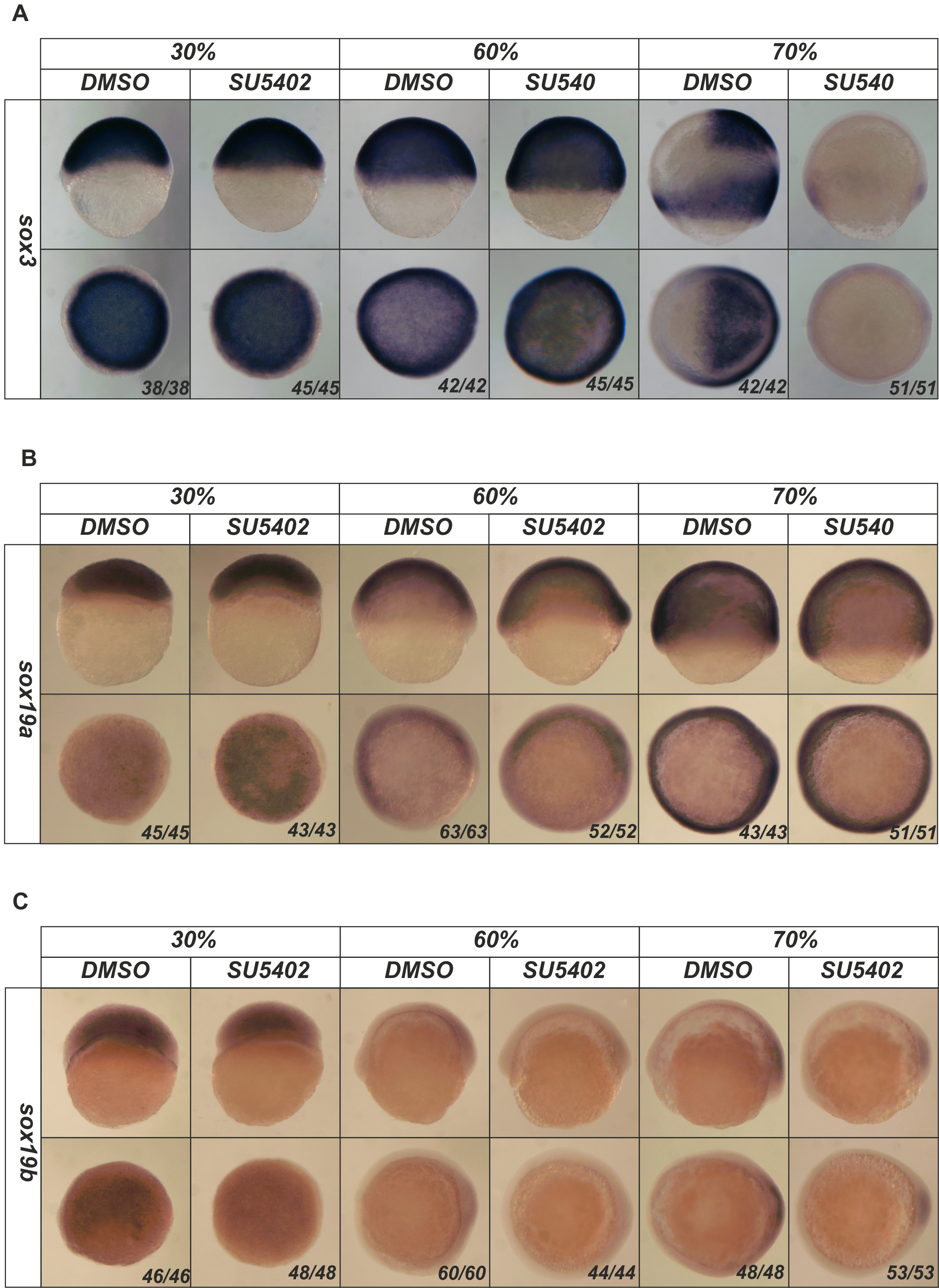

Supplement: Figure S10 — Expression of sox3, but not sox19a or sox19b, becomes Fgf-dependent between 60 and 70% epiboly. Embryos were treated with 84 µM SU5402 or DMSO alone from 3 hpf (the time that zygotic expression begins) and the expression of sox3 (A) sox19a (B) or sox19b (C) genes was analyzed at 30%, 60% and 70% epiboly. The expression patterns of sox3/19a/19b in embryos treated with SU5402 were the same as DMSO treated embryos at both 30% and 60% epiboly stage. At 70% epiboly, only sox3 expression was affected by SU5402, when it was strongly inhibited in embryos treated with SU5402. Lateral view and dorsal is to the right in upper panels, viewed from animal pole in lower panels. The proportion of embryos exhibiting these phenotypes is shown at the bottom right of each panel. (TIF) [file pone.0057698.s010.tif]

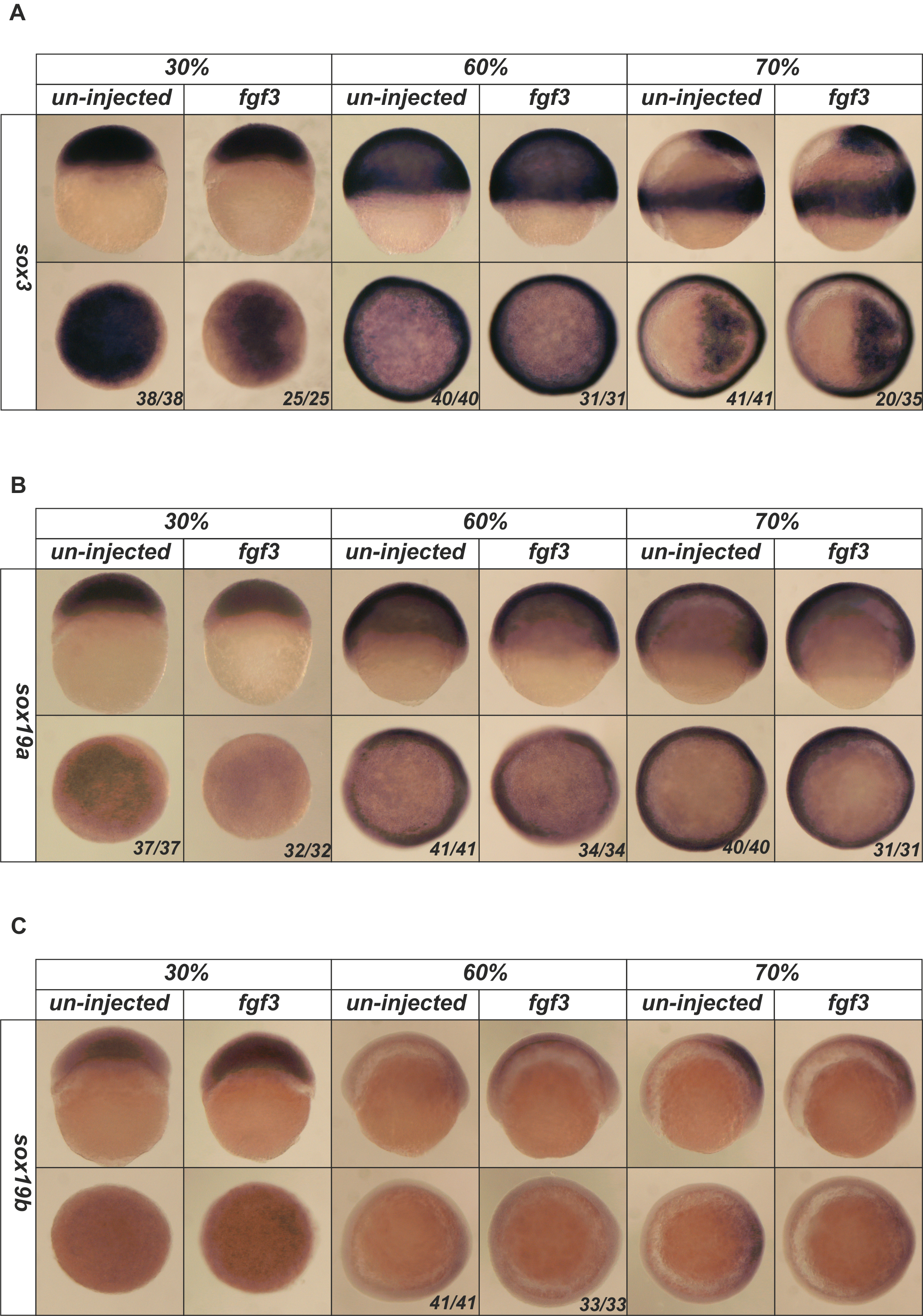

Supplement: Figure S11 — Over-expression of Fgf3 does not affect sox3/19a/19b expression. Embryos injected with 50 pg fgf3 mRNA at the 1–2 cell stage and the expression of sox3 (A) sox19a (B) or sox19b (C) was analysed at 30%, 60% and 70% epiboly. At no stage did injection of fgf3 RNA have any effect upon soxB1 gene expression. Lateral view and dorsal is to the right in upper panels, viewed from animal pole in lower panels. The proportion of embryos exhibiting these phenotypes is shown at the bottom right of each panel. (TIF) [file pone.0057698.s011.tif]
