# Supplementary material for: Formation of the Embryonic Organizer Is Restricted by the Competitive Influences of Fgf Signaling and the SoxB1 Transcription Factors
Source: PLoS One. 2013 Feb 28;8(2):e57698. doi: 10.1371/journal.pone.0057698 (PMC3585176; doi:10.1371/journal.pone.0057698)
Supplement: Figure S4 — Clustal alignments of genomic regions upstream of fgf3 across diverse species. (A–D as described in Fig. 2). Sox binding consensus sequences in gray boxes. Stars show bases entirely conserved in species shown. Numbering relates to the zebrafish genome relative to the fgf3 transcription start site at position 28694. (DOCX) [file pone.0057698.s004.docx]

**Region A**

Zebrafish 6018 ACATAAAACCCGCATTTCTCAGTTTTCAAATGTCTCATTG----AGGC-TCTCACAGGCTT 6067

Gar ACATAAAACCTGTGTTTCTCAGTTTTCAAATATCTTATTG----AGCC-TCTCATAG-CTT

Coelacanth ACATAAAACCAGTGTTACTCAGTTTTCAA-TGTCTTATTG----AGCCCTCTCACAG-CTT

Cichlid ACACAAAACCAATGTTTCTCAGTTTTCAGATGTCTCATTG----AAGGCTCGTTTGG-CTT Stickleback ACATAAAACCAGTGATTCTCAGTATTCAGATATCGCATTG--------------ACG-CTT

Chick ATATAAAACCTGTGTTACTCAGTTTTCAA-TATCTCATTG----AACT-TCTCACAG-CTT Zebrafinch ATATAAAACCTGTGTTACTCACTTTTCAA-TATCTCATTG----AACA-TCTCACAG-CTT

Turtle ATATAAAACCTCTGTTACTCAGTTTTCAA-TATCTCATTG----AGCC-TCTCACAG-CTT

Opossum TCACAAAACCTGGGGTTCTCTGCTTTCAA-TATGTCATTGTGTGGCCGC-CTCGCAG-CTT

Mouse TCACAGAACCTGGGTGACTCAGCTTTCAG-TGGGGCATTG-----TGGC-CTCCCAG-CTT

* * **** *** **** * **** * ***

Zebrafish 6068 TGATATTTTAAGTAGGTATCACAACAAAGGGGCA-ACAAATGG 6115

Gar TGATATTTTAAGTAGGTATCACAACAAAAGGACA-ACAAATGG

Coelacanth TGATATTTTAAGTAGGTATCACAACAAAGGGACA-ACAAATGG

Cichlid TGATATTTTAAGTAGGTATCACAAATAAAGGGCC-AATAGGA-

Stickleback TGATATTTTAAGTAGGTATCACAACAAAAGGGCT-TCACCTGG

Chick TGATATTTTAAGTAGGTATCACAACAAAGGGAAA-AGAAGTGA

Zebrafinch TGATATTTTAAGTAGGTATCACAACAAAGGGAAACAGAAGTGA

Turtle TGATATTTTAAGTAGGCATCACAACAAAGGGAAA-ACAAATGA

Opossum TGATATTTTAAGTCGGAATCCTAACAAAGCAAAG-ACAGATGA

Mouse TGATATTACAAGTGAGCGTCAAAACAAAGGGGCT-TCGAGGGG

******* **** * ** ** **

**Region B**

Zebrafish 24696 ACAACCATTGATGCCTT-ATACAGTTACAGGCCACCACAATCATTTTACTGTTGGAAGGT 24754

Gar ACAGTCACTGATGGCTT-ATACTGTTACAGGCCATCACAATCATTTTACTGTTGG--AGC

Coelacanth ACAGTCACCAACAAGATGATAGTGTTACAATCCATTACAAAGATTTTACTGTTGGAAAGT

Cichlid ACAGCCATTGATGGCCT-CTAGAGTTTCATCCTTCAACAATCACTTGCCTGTTGCAAGGT

Stickleback ACAGCCATTGATGGCCT-CTAGAGTTTCATCCTTCCACAATCATTTGCCTGTTGGAAGGT

Tetraodon ATAGCCATTGATGGCCT-ATTGAGTTTCATCCTTCCACAATTGCTTGCCTGTTGGAAGGT

Turtle ACCGTCACCAGGAGCCAGATTGTGTTACCCTTCATCACAAAGATTTCACTGTTGGAAAGT

* ** * *** * **** ** ****** *

Zebrafish 24755 GCAGAGAAATCCACTGTCCTCCCAAAAGGTATTTGAGACCTGCAGCTAAGAATCAAAGGG 24814

Gar ACAGGGTATTCTAATG-CCTTCTGAAAGGTATTTGAAACCTGAAGGCAGGAATCAAAGGG

Coelacanth GTAGAGCATTCTAATG-CTTTCTGAAAGGTATTTGAAACTTGAAGTCAGGAATCAAAGAG

Cichlid GCAGCCGCTCT-------------GCAGGCATATGAAACCTGCAGCTAACAATCAAAGAG

Stickleback GCAGCCACTCT-------------GCAGGCATCTGAGACCTGCAGCTAACAATCACTGAG

Tetraodon GCAGCCGCTTT-------------GGAGGTATCTGAAACCTGCTGCTAACAATCAAAGAG

Turtle GTAGAGTATTCTGATG-CTTTCTGAAAGCTATTTGAAATTTGAAGTCAAGAATCAAAGAG

** ** *** * ** * ***** * *

Zebrafish 24815 CTGCAGAGGTATAAGGAAGACCTATCTGGTTTCAAG 24850

Gar ATCTGAAGGTATAAGAAAG-CCTATCCAATTTCAAG

Coelacanth AGCTGAAAGTAGAAGAAAG-CCTATCCAATTTCAAG

Cichlid CTGTAGAAGTATAGGACAGACCTATCTAGTTACAGA

Stickleback CTGTAGGAGCATCAGACAGACCTCTCCGGTTTCACA

Tetraodon CTGTATGAGTATACGACAGACCTATCTGGTTACAGA

Stickleback CTGTAGGAGCATCAGACAGACCTCTCCGGTTTCACA

Turtle AGGTGAAAGTGTAAGAAAG-CCTATCCAGTTTCAAG

* * * ** *** ** ** **

**Region C**

Zebrafish 25510 GTTGCCAATTTGTTTGCAG---TGCG-TTGGGACTGTACGGGAGTTCTTTCTGAG-CCCC 25554

Gar GTTGCCAATTTGTTTGCAA---TGCG-GTCGAACTGTACGGGAGATCTTTTTGCC----T

Coelacanth GTTGCCAATTTGTTTGCAA---TGCTGTTCTAACTGTACGGGAGTTCTT-TTTC---TCC

Platyfish GTTGCTGATTTGTTTGCTA---TGTG-CTGGAACTGTACTAGACTTCTTTCTGAAGAGTC Stickleback GTTGCCAATTTGTTTGCTG---TGTG-CTGGAACTGTACGAGAGCTCTTTGTGAGGCGCC

Chick GTTGCCAATTTGTTTGCAA---TGCA-TTCTGACTGTACGGGAGTTCTT-TTTT---TCC

Zebrafinch GTTGCCAATTTGTTTGCAA---TGCA-TTCTGACTGTACGGGAGTTCTT-TTCT---GCC

Lizard GTTGCCAATTTGTTTGTAA---TGCA-TTCTGACTGTACGGGAGTTCTT-TTTAC--TCC

Turtle GTTGCCAATTTCTTTGCAA---TGCA-TTCTGACTGTACGGGAGTTCTT-TTTC---TCC

Tropicalis GTTGGCAATTTGTTTGCAG---TGCT-TTCTGCCTGCACCGGAGCTCTTGTTTTC-CCCC

Platypus GTTGCCAATTTGTTTGCAA---TGCG-TGGTGACTGTACAGGAGCCCTT-TTTC---TCC

Human GCTGTCAGTTTGCAGGCAGGTGTGGGACAGGGCCTAGGCAGGCTCTTTCCCTGGG-----

* ** ..*** : * :. ** ..**. .* .*. * *

Zebrafish 25565 TGACAAGCAGGTGAGATCAAAGTAAACCTGGACAAAAGGG----GCTCAACAGTGATTTG 25620

Gar TGACAGGCAGGTGAGATCAAAGAGCGAGGGGACAAAAGGC----GCTCAGCAGCGATTTG

Coelacanth TGACAGGCAGGTGAGATCAAAGTGGTGCAGGACAAAAGGG----GCTCAGCAGTGATTTG

Platyfish TGACAAGCAGGTGAGATCAAAGTCCTGG-GGACAAAAGAG----GCTCTGCAGCGATTTG Stickleback TGACAAGCAGGTGAGATCAAAGTCAGGG-GGACAAAAGAG----GCTCAGCAGCGATTTG

Chick TGACAGGCAGGTGAGATCAAAGTGGTGCAGGACAAAAGGG----GCTCAGCAGTGATTTG

Zebrafinch TGACAGGCAGGTGAGATCAAAGTGGTGCAGGACAAAAGGG----GCTCAGCAGTGATTTG

Lizard TGACAGGCAGGTGAGATCAAAGTGATGGAGGACAAAAGGG----GCTCAGCAGTGATTTG

Turtle TGACAGGCAGGTGAGATCAAAGTGGTGCAGGACAAAAGGG----GCTCAGCAGTGATTTG

Tropicalis TGACAAGCAGGTGAGATCAAAGCGACCCAGGACAAAGGGG----GCTCCACAGTGATTTG

Platypus TGACAGGCAGGTGAGATCAAAGAGGGGCAGTACAAAAGGGGCTGGCTCAGCAGTGATTTG

Human TGACAGGCAGGCAAGATCAAAAGCTGTTGGGACAAAAGGG----GCTGAG--GTGAATTG

*****.***** .********. * *****.*. *** . * **:***

Zebrafish 25621 TCAGGGGACGAGAGGCATTCCCGTCTG--------------GCTCTAGCCAGGCCCTCAG 25666

Gar TCAGGGGACGAAAGGCAGTCCTGTCTGGGAAAGTGGCTGCAGCCCCGGCCGGGCCCTCAG

Coelacanth TCAGGGGACGAAAGGCAGGCCTGTCTGGGAAAGTGGCTGTAGCCCAGACCGGGCCTTCAG

Platyfish TCAGGAGACGAGAGGCAG---CGGCTG--------------GATT----CAG-CCCTCTG Stickleback TCAGGAGACGAGAGGCAG---CGGCCG--------------GGTC----CAG-CCCTCTG

Chick TCAGGGGACGAAAGGCAGTCCTGTCTGGGAAAGTGGCTGTAGCCCAGAACGAGCCTTCAG

Zebrafinch TCAGGGGACGAAAGGCAGTCCTGTCTGGGAAAGTGGCTGTAGCCCAGAACGAGCCTTCAG

Lizard TCAGGGGACGAAAGGCAGTCTTGTCTGGGAAAGTGGCTGTAGCCCAGAACGATCCTTCAG

Turtle TCAGGGGACGAAAGGCAGTCCTGTCTGGGAAAGTGGCTGTAGCCCAGAACGAGCCTTCAG

Tropicalis TCAGGGGACGAAAGGCAGCGCTGTCTGGGAAATCAACTGCAGCCCAGAACGGGCCTTCAC

Platypus TCAGGGGACGAAAGGCA-TCCTGTCTGGGAAAGTGGCTGCAGCCCAGAACGGGCCTTCAG

Human TCCTGG--CCAAAGGCAGGGCACTCTGGGACAGTG--TGCAGCCCAGTCAGG--CCTCAG

**. *. * *.***** * * * ... * **:

Zebrafish 25667 ATCACTAGCGCAAAAAAAAAAAAAAAAAAAAAACGCCATGAGAAAAAGTACAGCAGAAAA 25726

Gar ATCACTGGCACACTAAAGAAATCGT---------GGCATGAGAAAAAGTACAGCAAAAAA

Coelacanth ATCACTAACACACTAAAGAAATCAT---------GGCATGAGAAAAAGTACAGCAGAAAA

Platyfish ATCACCGCCTCACTAAAGAAATCTG---------CCCATGAGAAAAAGTACAGCAGAAAG Stickleback ATCACCGCCCCACCAAAGAACTCTG---------TCCATGAGAAAAAGTACAGCAGAAAG

Chick ATCACTAACACACTAAAGAAATCAT---------GGCATGAGAAAAAGTACAGCAGAAAA

Zebrafinch ATCACTAACAGACTAAAGAAATCAT---------GGCATGAGAAAAAGTACAGCAAAAAA

Lizard ATCACTAACACGCTAAAGAAATCAT---------GGCATGAGAAAAAGTACAGCAGAAAA

Turtle ATCACTAACACACTAAAGAAATCAT---------GGCATGAGAAAAAGTACAGCAGAAAA

Tropicalis TTCAGCAGG--ACTAAAGAAATCGC---------GGCATGAGAAAATGTACAGCAGAAAA

Platypus ATCACTAACACACTAAAGAAATCAT---------GGCATGAGAAAAAGTACAGCAGAAAA Tropicalis TTCAGCAGG--ACTAAAGAAATCGC---------GGCATGAGAAAATGTACAGCAGAAAA

Human ACATGCACCCCAGAAGAACCCCCAC-----------CCTGACAGCAAG--CACCATGTGG

: .: . . *.*.... . *.*** *..*:* ** ** .:..

Zebrafish 25727 GGTTCAGAGGTCAG-CTTGATTTGGTCTGGATTGTG------------------CAAAAG 25767

Gar GGTTCAAAGGTCAG-TTTGATTTGGTTTGGATTATTGGTGCCTCCTTCCTTGGACAAAAG

Coelacanth GGTTCAAAGGTCAG-TTTGATTTGGTTTGGATTATTGGTGCCTCTTTT--AATGAAAGAG

Platyfish GGTTCAAAGGTCAT-TTTGATTTGCTCTGGATTACTGGTACCTCCTTTTCCCTCCATTAA Stickleback GGTTCAAAGGTCAT-TTTGATTTGCTCTGGATTACTGGTACCTCCTTTTTCCTCTGCTAA

Chick GGTTCAAAGGTCAG-TTTTATTTGGTTTGGATTATTGGTGCCTCCTTTTAAACCAAAGAG

Zebrafinch GGTTCAAAGGTCAG-TTTTATTTGGTTTGGATTATTGGTGCCTCCTTTCAAATCAAAGAG

Lizard GGTTCAAAGGTCAG-TTTTATTTGGTTTAGATTGTTGGTGCCTCCTTTTCAACCACAGAC

Turtle GGTTCAAAGGTCAG-TTTTATTTGGTTTGGATTATTGGTGCCTCCTTTTAAACCAAAGAG

Tropicalis GGTTCAAAGGTCAGGGTTGATTTGGTTCGGATTATAGGAGCCTCCTTT--------TAAA

Platypus GGTTCAAAGGTCAG-TTTTATTTGGTTTGGATTATTGGTGCCTCCTTTCAAACCAGAGTA

Human GGGTCAAAGGTCGG--GGTGTTTGGCTTGGATTACGG-TGCCTGCCTTTCACCCAGAGCA

** ***.*****. .**** .****. *

Zebrafish 25768 AACCAG----------AAAAAAGACTAG----TGACTATTAAGCTTTGTTGAAGGGGGAG 25816

Gar AGCCAA----------GAAAAAGAACCG---ATGGCTAATAACCTTTGTAATCTTG-GAA

Coelacanth CAAAAAGAA---------TAAGGAATAG------ACCAATAACCTTCACTGTTATACATA

Platyfish AAGAGG---------GAGCCAAGAAAAGGGCCTTGCTAATAACATTTGTATTATTGCAGA Stickleback AAGAGGGCTGGGGGCGGGGCAAGAAAAGGACCTGGCTAATAACCTTTGTATTATTGCAGA

Chick CAAGAAGGG------GAAAAAAGAACAG------GCTAATAACCTTTGTTATTGTCCAGG

Zebrafinch CAAGAAGGGG-----GAAAAAAGAACAG------GCTAATAACCTTTGTTATTGTCCAGG

Lizard CAAGAGGGG--------GGAAAGAACAG------GCTAATAACTGCTATTATTGTCCAGG

Turtle CAAGAGGG---------AAAAAGAACAG------GCTAATAACCCTTGTTATTGTCCAGG

Tropicalis CCTGAG-------------AAAGAACAG------GATAATAACCTTTGTTAATGTCCGGG

Platypus --AGAGAGG---------AAAAGAACTG------GCTAATAACCTTTGTTACTGGGGGGA

Human AGAAAG-----------CCAGAGAGCAG------GATAAGAACCTTCCGGATTGGAACTG

.. ... *: ** .. .

**Region D**

Zebrafish 27881 CCAAA-GGGCAGGGAGATTTGTAGATGATGGTGCTAATTACGAGAATGGTAACCA-TAAT 27898 Coelacanth CCCAA--GGCAGAGAGATTTGTAGATGATGGTGCTAATTATGAAAATGGTAACCA-TAAT

Gar CCAAA-GGGGAGAGAGATTTGTAGATGATGGTGCTAATTATGAAAATGGTAACCA-TAAC

Cichlid CCAAA-GGGCAGAGAGATTTGTAGATGATGGTGCTAATTACCAGAATG-AAACCA-TAAT

Platyfish CCAAA-GGGCAGAGAGATTTGTAGAAGATGGTGCTAATTACCAGAATGCAAACCA-TAAT

Stickleback CCAAA-GGGCAGGGAGATTTGTAGATGATGGTGCTAATTACCAGAATGCAAACCA-TAAT

Tetraodon CCAAA-GGGCAGAGAGATTTGTAGACGATGGTGCTAATTACCAAAATGCAAACCA-TAAT

Tropicalis CCAAA-GGCACAAGGGCTTTGAAA-TGATGGCGCTAATTATGAAAATAGCAAGGAGTAAT

Chick CCCA--GGGCGGAAAGATTTGTAGATGATGGTGCTAATTATGAAAATG------------

Turkey CCCG--GGGCGGAAAGATTTGTAGATGATGGTGCTAATTATGAAAATG------------

Zebrafinch CCCA--GGGCGGAAAGATTTGTGGCTGATGGCGCTAATTATGAAAATG------------

Turtle CCCA--GGGCGGAAAGATTTGTAGATGATGGTGCTAATTATAAAAATGGTAACCA-TAAT

Opossum CCCAAAGGCAGCAGCGCTGGGGAGATGATGGCGGTAATTAGCAAAATCGTCCCCA-TAAT

Platypus CCCAGAGGCG-AAGAGTTGGGGCGATGATGGGGCTAATTAGGAAAATAGGAACCA-TAAT

** ** * * ***** * ****** ***** *

Zebrafish 27899 GCGACCAAGATGGAAGCAATTTAGGCAGAAGTCATTTG 27976

Coelacanth GTTACCAAGATGGAAGCAATTTGAGCAG-AGTCGTTTG

Gar GCGACCAATATGGAAACAATTTAAGCAAAAGTCATTTG

Cichlid GTGACCAAGATGGAAGCAATTTAGGCAGTAGTCATTTA

Platyfish GTGACCAAGATGGAAGCAATTTAGGCAGTAGTCATTTA

Stickleback GCGACCAAGATGGAAGCAATTTAGGCAGTAGTCATTTA

Tetraodon GTGACCAAGATGGAAGCAATTTACGCAGCAGTCATTTA

Tropicalis GTTACCAAGATGGAAGCAATTTGGGCAAAAGTCATTTG

Chick -TGATCAAGATGGAAGCAGTTTGAGCAGAAGTCATTAA

Turkey -TGATCAAGATGGAAGCTATTTGAGCAGAAGTCATTAA

Zebrafinch -TGATCAAGATGGAAACAATTTGAGCAAAAGTCATTAG

Turtle GTGATCAAGATGGAAGCAATTTGAGCAAAAGTCATTAA

Opossum GGGAGCAAGCTGGAAGCAATTTGCGGAGCAGTTATTAA

Platypus GCCACCAAGATGGAAGCAATTTCAGCCGAAGTTATTAA

* *** ***** * *** * *** **
